# Vegetable and Fruit Intake Variety and Cardiovascular Health and Mortality: A Systematic Review and Meta-Analysis of Observational Studies

**DOI:** 10.3390/nu15234913

**Published:** 2023-11-24

**Authors:** Stephanie K. Nishi, Nadine Khoury, Cristina Valle Hita, Andreea Zurbau, Jordi Salas-Salvadó, Nancy Babio

**Affiliations:** 1 Universitat Rovira i Virgili, Departament de Bioquímica i Biotecnologia, Unitat de Nutrició Humana, Grup Alimentació, Nutrició, Desenvolupament i Salut Mental, 43201 Reus, Spain; nadine.alkhoury@iispv.cat (N.K.); cristina.valle@urv.cat (C.V.H.); jordi.salas@urv.cat (J.S.-S.); 2 Institut d’Investigació Pere Virgili (IISPV), Carrer Dr. Mallafré Guasch, 4, 43007 Tarragona, Spain; 3 Consorcio CIBER, M.P. Fisiopatología de la Obesidad y Nutrición (CIBERobn), Instituto de Salud Carlos III (ISCIII), 28029 Madrid, Spain; 4 Toronto 3D Knowledge Synthesis and Clinical Trials Unit, Clinical Nutrition and Risk Factor Modification Center, St. Michael’s Hospital, Toronto, ON M5C 2T2, Canada; andreea.zurbau@mail.utoronto.ca; 5 Department of Nutritional Sciences, Temerty Faculty of Medicine, University of Toronto, Toronto, ON M5S 1A8, Canada

**Keywords:** vegetable variety, fruit variety, cardiovascular disease, stroke, mortality, grade approach

## Abstract

Introduction: A multitude of evidence supports the consumption of a higher quantity of vegetables and fruits for their cardiovascular benefits. Nonetheless, the extent to which variety is associated with cardiovascular health remains unclear. Objective: To conduct a systematic review and meta-analysis of observational studies (prospective cohort and cross-sectional studies) assessing the role of a variety of vegetable and fruit consumption in cardiovascular morbidity and mortality in adults. Data Sources: MEDLINE-PubMed, Cochrane databases, and reference lists were searched through March 2023. Data Extraction: Two independent reviewers extracted data and assessed the risk of bias (National Heart, Lung, and Blood Institute Tool and Newcastle–Ottawa Scale). Data Analysis: Data were pooled (fixed and random [DerSimonian and Laird] effects for <5 and ≥5 study comparisons, respectively), and heterogeneity was assessed using the Cochran Q statistic and quantified (I^2^ statistic). The Grading of Recommendations, Assessment, Development, and Evaluation (GRADE) was used to assess the overall certainty of the evidence. Five cross-sectional (n = 45,761) and seven prospective studies (n = 253,422) met the eligibility criteria. Greater variety of vegetable and fruit consumption was prospectively related to decreased all-cause mortality (risk ratio, 0.89 [95% CI, 0.82–0.97], seven study comparisons, n = 196,925), while no significant associations were observed with assessed cardiovascular-related mortality or morbidity. For all outcomes, the certainty of the evidence was graded as “low” or “very low” owing to inconsistency and/or imprecision. Conclusions: Overall, this study shows that greater variety in vegetable and fruit consumption may reduce all-cause mortality and highlights the need for additional studies with a higher degree of evidence to better understand its role in cardiovascular health.

## 1. Introduction

Cardiovascular diseases have collectively remained the leading causes of morbidity and mortality worldwide. Over 55 million people have experienced cardiovascular disease, and it has been associated with 32% of mortality globally [1]. Of these deaths, 85% are attributed to myocardial infarction and stroke, and of all the premature deaths occurring due to noncommunicable diseases, 38% were caused by cardiovascular diseases (CVD) [2]. Lifestyle factors, such as diet, have been implicated in cardiovascular health. In 2021, it was estimated that dietary-related risks accounted for almost 7 million cardiovascular deaths and 8 million deaths [1].

Plant-based dietary patterns, such as the Mediterranean diet, have been associated with many health benefits [3,4,5,6]. While the definition of what constitutes a plant-based dietary pattern can vary greatly depending on the extent to which a person excludes animal products from their daily intake, the basic principles focus on the consumption of plants, of which vegetables and fruits comprise a significant component. There is a wealth of evidence endorsing the intake of a larger quantity of vegetables and fruits due to their cardioprotective qualities [7]. An adequate intake of vegetables and fruit has been associated with a lower risk of CVD, but also cancer and all-cause mortality [8,9]. Dietary guidelines around the world, including those in Australia, Europe, and North America, all recommend increasing vegetable and fruit variety to increase both the quantity and diversity of the nutrients consumed [10,11,12]. “Variety” is a term broadly used in dietary guidelines [12]; still, it is not always explicitly specified. There is a lack of clear evidence regarding the connection between the diversity of fruits and vegetables in one’s diet and its impact on health. This knowledge could potentially shape future strategies for population dietary interventions and health policies. Furthermore, a health claim proposal relating to vegetables and fruits and cardiac function had previously been submitted to the European Food Safety Authority (EFSA); however, the EFSA Panel noted that the variety and amount of the specific vegetables/fruits required to obtain the claimed effects were lacking [13]. Stronger evidence, such as that from a systematic review and meta-analysis, is needed to support and inform specific recommendations when it comes to variety (e.g., diversity), over and above total quantity, of vegetable and fruit consumption with cardiovascular health.

To date, to our knowledge, no systematic review and meta-analysis has been conducted to assess and update the evidence on which cardiovascular-related recommendations and public health policy regarding variety in vegetable and fruit intake are based. Thus, a systematic review and meta-analysis were conducted to assess the role of a variety of vegetable and fruit consumption and CVD risk, morbidity, and mortality in observational studies, including an appraisal of the certainty of the evidence using the Grading of Recommendations, Assessment, Development, and Evaluation (GRADE) approach.

## 2. Methods

### 2.1. Study Design

The methodological guidelines of the Cochrane Handbook for Systematic Reviews of Interventions [1] were followed, and results were reported in accordance with the Preferred Reporting Items for Systematic Reviews and Meta-Analyses statement (PRISMA) [2] and the Meta-Analysis of Observational Studies in Epidemiology (MOOSE) guidelines [3] (Supplementary Tables S1 and S2). The protocol was registered on PROSPERO with the identifier CRD42041262911.

### 2.2. Eligibility

Supplementary Table S3 provides the PECOTS (population, exposure, comparator, outcome, timeline, and setting/study design) framework. PECOTS was used to develop the question being addressed. Of interest were adults (aged ≥ 18 years) in the general population free of CVD at baseline in the prospective cohort studies, who were not breastfeeding or pregnant (P), who had higher variety in vegetable and/or fruit intake (E), and were compared to participants with dietary patterns with lower variety of vegetable and/or fruit intakes (C). Incidence and prevalence of CVD, coronary heart disease (CHD), and stroke, as well as CVD-related and all-cause mortality, were the main outcomes of interest (O), with CVD risk factors, including lipids, blood pressure, glucose control, inflammation, body composition, and risk scores as secondary outcomes. Full-text reports conducting a cross-sectional (one time point) or prospective cohort (at least 1 year in duration) study design (T and S) were included. Studies were excluded if they did not meet the above criteria. No constraints were placed on article language.

### 2.3. Data Sources, Search, and Screening

MEDLINE-PubMed and Cochrane Library databases were comprehensively searched from their inception until 21 March 2023. Relevant MeSH terms and keywords were used. Supplementary Table S4 depicts the detailed search terms and strategy. Additionally, a manual review of the reference lists of the acquired articles was conducted to ascertain the identification of all pertinent studies. The references were then uploaded into the online screening program, Abstrackr (http://abstrackr.cebm.brown.edu/), where two independent reviewers (NK and CVH) carried out an initial screening of the titles and abstracts of the obtained papers for eligibility. Subsequently, the full texts of potentially eligible studies underwent a duplicate review for inclusion. Any disparities were resolved through discourse with a senior researcher (SKN and AZ) and consensus. Cross-sectional and prospective studies (with ≥1 year of follow-up) evaluating the association between dietary vegetable and/or fruit variety and cardiovascular-related risk, morbidity, mortality, all-cause mortality, and CVD-related risk factors in adults (aged ≥ 18 years) were included. When study data had been published on more than one occasion, the report with the longest follow-up duration was included unless the publications assessed different relevant outcomes; then, each report presenting findings related to unique, relevant outcomes was included.

### 2.4. Data Extraction

Three independent reviewers (CVH and NK or SKN) extracted pertinent information from the studies meeting all eligibility criteria. This included details such as author, year, journal of publication, country, study name, study design, total sample size, duration of follow-up (relevant only for prospective cohort studies), participant characteristics, exposure(s) and method of assessment, outcome(s) and method of assessment, number of cases (for morbidity and mortality outcomes), funding sources, statistical analyses conducted, and multivariable-adjusted effect estimators (e.g., mean differences and standard deviations, beta-coefficients, odds ratios, risk ratios, or hazard ratios and 95% confidence intervals) for the associations under investigation. A third researcher (SKN and AZ or JSS) was consulted to solve any disagreements. All data were extracted using an electronic spreadsheet. Authors were contacted in cases where relevant outcome data were missing when it was indicated that it was measured but not reported, or data were not provided in a way that could be appropriately meta-analyzed. When outcome data were only presented graphically, Plot Digitizer was used to extract information [4].

### 2.5. Outcomes

The main outcomes of interest of the present systematic review and meta-analysis encompassed cardiovascular-related morbidity and mortality, as well as all-cause mortality. Specifically, morbidity and mortality-related outcomes of interest included prevalence (in cross-sectional studies) and incidence (in prospective cohort studies) of CHD, stroke, and overall CVD, as well as incidence of cardiovascular and total mortality from reports or medical records. Cardiovascular-related risk factors were also assessed.

Cardiovascular risk factors of interest included blood lipids (e.g., low-density lipoprotein-cholesterol [LDL-C], high-density lipoprotein-cholesterol [HDL-C], triglycerides [TG], and total cholesterol [TC]), blood pressure (systolic [SBP] and diastolic [DBP]), body composition (e.g., body weight, waist circumference, and body mass index [BMI]), inflammation (e.g., C-reactive protein), and glucose control (e.g., fasting blood glucose, homeostatic model assessment of insulin resistance [HOMA-IR], and hemoglobin A1c [HbA1c]) factors, and cardiovascular risk scores (e.g., 10-year cardiovascular risk as determined by the Framingham Risk Score [FRS]).

### 2.6. Quality Assessment

Two independent reviewers (CHV and NK) assessed the study quality via an appraisal of the potential risk of bias in each of the included studies. Two distinct tools for evaluating study quality were employed. The Study Quality Assessment Tool for Observational Cohort and Cross-Sectional Studies from the National Heart, Lung, and Blood Institute [5] was utilized for cross-sectional studies. This tool comprises 14 questions designed to guide assessors in summarizing the overall quality of the study, categorized as either poor, fair, or good.

In the case of prospective cohort studies, the Newcastle–Ottawa Scale (NOS) [6] was employed. This scale assigns a rating ranging from 0 to 9 points to studies, evaluating them across three domains: (1) population selection, (2) outcome assessment, and (3) comparability. Studies with a total score of at least 7 points were classified as high quality (indicating low risk of bias), a score of 6 was deemed moderate, and a score of ≤5 was adjudged as low study quality.

In cases of disagreement among researchers, resolution was sought through consulting senior researchers (SKN) and via consensus.

### 2.7. Data Synthesis and Analysis

All analyses were conducted using Stata software, version 17.0 (StataCorp LP, College Station, TX, USA), and Review Manager version 5.4 (The Nordic Cochrane Centre, Copenhagen, Denmark).

The logarithmically transformed odds ratios (ORs), risk ratios (RRs), and hazard ratios (HRs), along with their corresponding 95% CI comparing highest versus lowest (reference) categories of dietary vegetable and/or fruit variety consumed, were pooled using the generic inverse variance method. Fixed-effects models were utilized for fewer than five comparisons and random-effects models for at least five comparisons. Distinct meta-analyses were carried out for cross-sectional and prospective cohort studies. HRs and ORs (as cumulative incidence <10%) were considered equivalent to RR [7,8]. For open-ended lower and upper quantiles, the lowest and highest boundary was defined as the same as the adjacent category cutoff. Correlations were converted to standardized mean differences [9].

The presence of heterogeneity was evaluated using the Cochran Q statistic and quantified through the I^2^ statistic. Substantial heterogeneity was deemed present if I^2^ was ≥50% and the *p*-value for heterogeneity was less than 0.10 [10,11].

Sensitivity (influence) analyses, examining the impact of individual studies, were performed in cases where there were more than three study comparisons. This involved systematically excluding one study at a time (using a leave-one-out approach) from the meta-analyses and then recalculating the summary risk estimates. A study was considered influential if exclusion resulted in a change of more than 20% in the evidence of heterogeneity, magnitude, significance, and/or direction of the association.

Significant unexplained heterogeneity was planned to be explored using a priori subgroup analyses if there were at least 10 studies, by sex, geographical area, underlying health status, assessment of vegetable and fruit intake, vegetable and fruit amount, follow-up duration (only for prospective cohorts), macronutrient intake, and risk of bias. Meta-regression was intended to evaluate the significance of subgroup analyses. Linear and nonlinear dose-response analyses were to be examined using generalized least squares trend (GLST) estimation models, suitable for weighted regression of summarized dose-response data with dependent components such as the reference exposure level. Additionally, spline curve modeling (utilizing the MKSPLINE procedure) would be considered where feasible. To examine publication bias, funnel plots were visually inspected, and Egger’s and Begg’s tests were applied, contingent upon having a minimum of 10 studies. If any asymmetry in the funnel plot was observed, adjustments would be made using the Duval and Tweedie trim-and-fill method.

### 2.8. Grading the Evidence

The GRADE approach was used to assess the certainty of the evidence by two independent reviewers (CVH and NK), with any disagreement resolved by consulting senior researchers (SKN and AZ) and via consensus. Due to their inherent limitations, observational studies initially receive a “low” certainty of the evidence based on a scale involving four possibilities ranging from “very low” to “high”, which may then be downgraded or upgraded based on established criteria [12]. The magnitude of the association was determined according to predefined criteria utilizing MIDs [14,15,16,17,18,19,20,21,22,23,24,25] and adapted GRADE thresholds, employing terminology such as “trivial”/”unimportant” (<1 MID), “small important effect” (≥1× MID), “moderate” (≥2× MID), and “large” (≥5× MID)’ [13,26,27]. 

## 3. Results

### 3.1. Search Results

**Figure 1:**
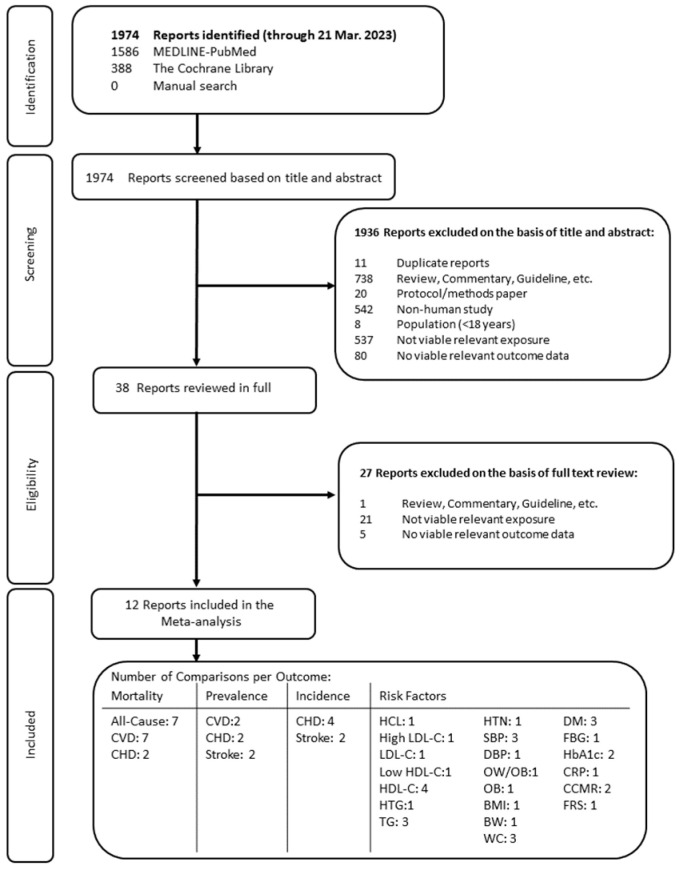
Summary of evidence search and selection. Abbreviations: BMI, body mass index; BW, body weight; CCMR, clustered cardiometabolic risk scores; CHD, coronary heart disease; CRP, C-reactive protein; CVD, cardiovascular disease; DBP, diastolic blood pressure; FBG, fasting blood glucose; FRS, Framingham risk score; HbA1c, glycated hemoglobin A1c; HCL, hypercholesterolemia; HDL-C, high-density lipoprotein-cholesterol; HTG, hypertriglyceridemia; HTN, hypertension; LDL-C, low-density lipoprotein-cholesterol; OB obesity; OW, overweight; SBP, systolic blood pressure; TG, triglycerides; WC, waist circumference.

Figure 1 illustrates the results of the literature search. Following an initial search of MEDLINE-PubMed and Cochrane databases, complemented by manual searches, a total of 1974 articles was identified. Of the authors who attempted to be contacted, additional relevant data were not available [28,29,30,31]. Of the 38 reports reviewed in full, 12 unique publications involving 5 cross-sectional and 7 prospective cohorts (14 cohort comparisons) met eligibility criteria.

### 3.2. Study Characteristics

Table 1 summarizes the characteristics of the included studies. Supplementary Tables S5 and S6 present the characteristics of each of the individual studies found eligible for inclusion. Study sizes ranged from 98 to 79,904 participants, including men and women. Most participants were middle-aged adults, with ages ranging from 18 to 98 years (median 46.8 years). Participants were from seven countries (China [1], Japan [1], the Netherlands [1], Spain [1], the United Kingdom [1], and the United States [7]). The prospective cohort durations ranged from 1 to 24 years (median 12 years). Morbidity and mortality were ascertained by medical records, death certificates, or equivalent (five cohorts) [32,33,34,35,36] or self-report (one cohort) [37], and CVD risk factors were determined by direct measurement and blood samples, with relevant calculations for LDL-C (e.g., Friedewald) and risk scores (e.g., Framingham risk score). Diet was measured using food frequency questionnaires [30,32,34,35,36,38,39,40], 24 h recall [33,37], or food records [29]. Supplementary Table S7 describes how vegetable and fruit variety was determined in each included study. Variety in vegetable and/or fruit intake was defined as the number of different types of produce consumed, and this may be reported based on differences in produce classifications or differences in specific produce. The majority of the studies received funding from an agency [33,34,35,36,37,39,40], and one study received partial funding through a mix of agencies and industry [29]; however, the authors maintained that the sponsors had no role in the study or its publication. Two studies did not report receiving any funding [32,38].

Supplementary Tables S8 and S9 present the covariate adjustments in the cross-sectional and prospective cohort studies, respectively. All the studies considered in the analyses controlled for the predetermined primary confounding variable of energy intake. Additionally, 10 studies adjusted for a minimum of four out of six important secondary confounding variables: age, sex, baseline amount of vegetable/fruit intake, physical activity, smoking, BMI, or body weight.

### 3.3. Study Quality Assessment

Supplementary Table S10a,b show a summary of the risk of bias (ROB) assessments of the included cross-sectional and prospective cohort studies, respectively. Most cross-sectional studies were assessed as having a fair quality rating and prospective cohort studies as having relatively high quality with NOS ≥ 6, based on their risk of bias determinations. Thus, there was no overall concern about the risk of bias in most study comparisons.

### 3.4. Variety in Vegetable and Fruit Consumption and Mortality

**Figure 2:**
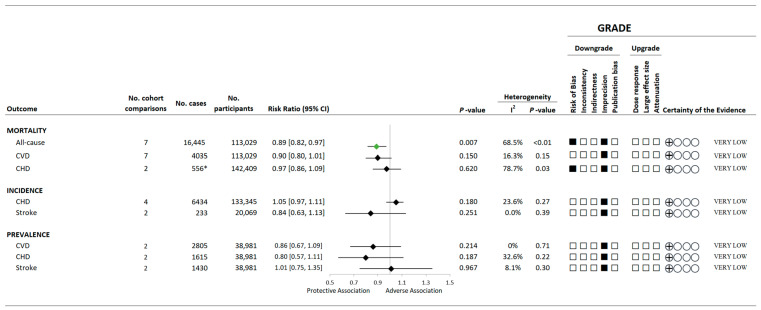
A summary plot for the association of a variety of vegetable and/or fruit intake with CVD-related incidence, prevalence, and mortality. Analyses were conducted using generic, inverse variance random-effects models (at least five study comparisons available) or fixed-effects models (fewer than five study comparisons available). Any statistically significant reductions in risk are denoted by a green diamond, whereas a black diamond denotes a lack of statistical significance. The GRADE for observational studies is rated as “low” certainty of evidence and can be downgraded by five domains and upgraded by three domains. The white squares represent no downgrade. The black squares indicate a single downgrade or upgrade for the respective domain. * One of the two eligible studies [32] did not report cases; thus, the number of cases could be equal to or greater than the presented number. Abbreviations: CHD, coronary heart disease; CI, confidence interval; CVD, cardiovascular disease; GRADE, Grading of Recommendations Assessment, Development and Evaluation approach; RR, risk ratio.

Figure 2 and Supplementary Figures S1–S3 present the summary and individual forest plots showing the associations of a greater variety of vegetable and fruit consumption with the assessed mortality outcomes. Overall, higher variety of vegetable and fruit consumption was associated with 11% reduction in all-cause mortality (7 comparisons, n = 113,029 total population, 16,445 cases; RR: 0.89, 95% CI: 0.82 to 0.97, *p* = 0.007; substantial heterogeneity: I^2^ = 68.5%, P_Q_ < 0.01; Supplementary Figure S2). However, non-significant associations were observed between a variety of vegetable and fruit consumption and CVD-specific mortality or CHD-related mortality. Viable data were not available to assess the relationship between the variety of vegetable and/or fruit intake and stroke-related mortality. Evidence of substantial inter-study heterogeneity was found for all-cause and CHD-related but not CVD morality.

### 3.5. Variety in Vegetable and Fruit Consumption and CVD Morbidity

Figure 2 and Supplementary Figures S4–S8 present the associations between higher intakes of vegetable and/or fruit variety and the incidence and prevalence of cardiovascular-related events. Generally, no overall associations were observed between the variety of vegetable and/or fruit intake and the incidence of CHD or stroke nor the prevalence of CVD, CHD, or stroke.

### 3.6. Variety in Vegetable and Fruit Consumption and CVD Risk Factors

**Figure 3:**
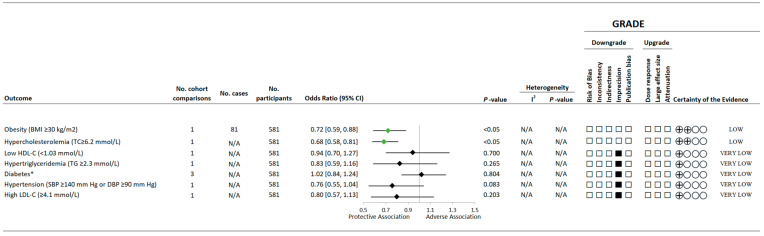
A summary plot for the association of a variety of vegetable and/or fruit intake with health statuses related to CVD risk. Analyses were conducted using generic inverse variance fixed-effects models (fewer than five study comparisons available). Any statistically significant reductions in risk are denoted by a green diamond, whereas a black diamond denotes a lack of statistical significance. The GRADE for observational studies is rated as “low” certainty of evidence and can be downgraded by five domains and upgraded by three domains. The white squares represent no downgrade. The black squares indicate a single downgrade or upgrade for the respective domain [13,26,41]. Any statistically significant reductions in risk are denoted by a green diamond, whereas a black diamond denotes a lack of statistical significance. * Type 2 diabetes in one study [30] was defined at FBG ≥ 7.0 mmol/L or 2HPG ≥ 11.1 mmol/L. Abbreviations: BMI, body mass index; CI, confidence interval; DBP, diastolic blood pressure; GRADE, Grading of Recommendations Assessment, Development and Evaluation approach; LDL-C, low-density lipoprotein-cholesterol; N/A, not available; SBP, systolic blood pressure; TC, total cholesterol; TG, triglycerides.

**Figure 4:**
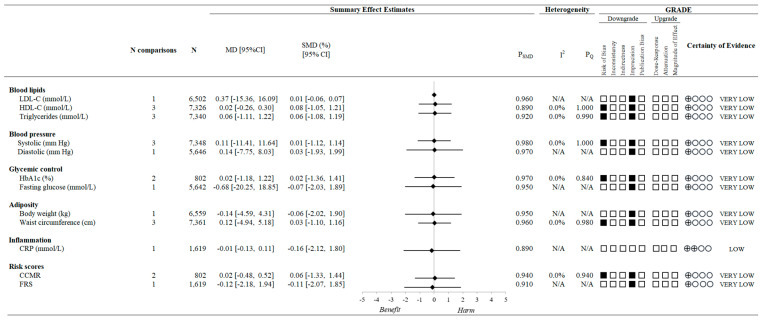
A summary plot for the association of a variety of vegetable and/or fruit intake with CVD risk factors. Analyses were conducted using generic, inverse variance random-effects models (at least five study comparisons available) or fixed-effects models (fewer than five study comparisons available). A black diamond denotes a lack of statistical significance. The GRADE for observational studies is rated as “low” certainty of evidence and can be downgraded by five domains and upgraded by three domains. The white squares represent no downgrade. The black squares indicate a single downgrade or upgrade for the respective domain [13,26,41]. Abbreviations: CCMR, clustered cardiometabolic risk scores; CI, confidence interval; CRP, C-reactive protein; FRS, Framingham risk score; GRADE, Grading of Recommendations Assessment, Development and Evaluation approach; HbA1c, hemoglobin A1c; HDL-C, high-density lipoprotein-cholesterol; LDL-C, low-density lipoprotein-cholesterol; MD, mean difference; SMD, standardized mean difference.

Figure 3 and Figure 4 and Supplementary Figures S9–S27 present the associations of the variety of vegetable and/or fruit consumption and CVD risk factors. Based on cross-sectional data, higher variety in vegetable and fruit consumption was associated with a 28% (95% CI 0.59 to 0.88, *p* < 0.05) lower risk of obesity and 32% (95% CI 0.58 to 0.81, *p* < 0.05) lower risk of having hypercholesterolemia but was not shown to be associated with elevated LDL-C, reduced HDL-C, hypertriglyceridemia, type 2 diabetes, or hypertension. Based on data from the prospective cohort studies, there were generally no overall associations observed between variety of vegetable and/or fruit intake and measures of blood lipids (LDL-C, HDL-C, and triglycerides), blood pressure (systolic and diastolic), glycemic control (HbA1c and fasting glucose), adiposity (body weight and waist circumference), inflammation (C-reactive protein [CRP]), or cardiovascular-related risk scores (clustered cardiometabolic risk scores [CCMR] and Framingham risk score [FRS]).

Due to the nature of the data, findings from two cross-sectional studies could not be meta-analyzed. Findings from these studies showed that a higher intake of total varieties of vegetables/fruit correlated with lower odds of overweight/obesity [41]. Additionally, an inverse relationship was observed between a 3-day fruit and vegetable variety score and BMI in women, very low-density lipoprotein and triglyceride levels in men, and a positive association with HDL-C in men (all *p* < 0.05) [29].

### 3.7. Sensitivity and Subgroup Analyses

Supplementary Figures S28–S35 show the sensitivity analyses. Systematic removal of each cohort comparison did not alter the significance or direction of the association between a variety of vegetable and/or fruit consumption and assessed CVD morbidity and risk factors, where applicable. However, in the analysis of a variety of vegetable and fruit consumption and CVD mortality, the removal of the study comparison of vegetable variety intake by men by Kobayashi and colleagues [34] resulted in a significant risk reduction, and heterogeneity was reduced to 0%.

A priori sub-group analyses were not carried out due to the insufficient number of studies (less than 10) available for each outcome. Similarly, meta-regression analyses for dose response were not pursued due to the limited number of studies with applicable data available.

### 3.8. Publication Bias

Potential publication bias could not be confidently tested since <10 study comparisons were included in each analysis [1].

### 3.9. Certainty of the Evidence

Figure 1 and Figure 2 and Supplementary Table S11 show the GRADE assessment. The certainty of evidence was considered “low” to “very low” for all assessed outcomes owing to the observational study design as well as downgrades for inconsistency and/or imprecision.

Interpretation of the magnitude of the impact indicated no effect for the majority of outcomes assessed, with a possible trivial impact of a variety of vegetable and fruit consumption in relation to all-cause mortality, hypercholesterolemia, and obesity risk.

## 4. Discussion

To our knowledge, this is the inaugural systematic review and meta-analysis of observational studies assessing the impact of vegetable and fruit variety consumption on mortality, morbidity, and risk related to cardiovascular health. Synthesis involving 113,029 participants with 16,445 events of death with a median follow-up of 14 years suggests a potential role for variety, independent of quantity, of vegetable and fruit consumption in reducing mortality risk. Findings also highlight the limited evidence and variation in methodologies currently present in the literature assessing a variety of vegetable/fruit intake in relation to morbidity and risk of CVD.

### 4.1. Findings in Relation to the Existing Literature

The present findings build on previous studies that have shown consumption of different individual vegetable and fruit intakes to be related to reduced incidence of cardiovascular outcomes [42] and mortality [43]. Compelling evidence has previously shown that a diet rich in vegetables and fruits from a quantity perspective can lower the risk of CVD, specifically heart disease and stroke. For instance, over 250,000 individuals over a median follow-up of 11–13 years who ate more than five servings of vegetables and fruit per day had a 17% reduction in CHD risk [44] and a 26% reduction in the risk of stroke [45], compared with individuals who ate less than three servings per day. Moreover, a Fruit and Vegetable Index (FAVI), which considers both the amount and variety of vegetables and fruits, has been inversely associated with weight gain, and hence CVD risk, over 6 years; however, the amount was small and likely not clinically significant [46]. Whereas the present findings suggest variety, apart from the amount, may also be associated with reduced obesity risk, evidence is currently limited and unclear given the few studies and the lack of association observed with body weight.

Vegetables and fruits, in general, are nutrient-rich; however, different varieties have different qualities, and the various combinations and concentrations of nutrients and bioactive components, for instance, micronutrients, phytochemicals, and fiber content, that they contain could possibly lead to different health benefits [47]. Indeed, dietary diversity, such as illustrated by increased fruit and vegetable variety, has long been recognized as a key component of diet quality [48]. Further, in men and women free of disease, higher consumption of a variety of vegetables and fruits has been related to lower total energy intake and higher dietary intakes of fiber, vitamins A and C, potassium, and magnesium [28,29]. Mechanistically, bioactive components of vegetables and fruit, such as vitamin C, carotenoids, polyphenols, potassium, and fiber, have been proposed to support a beneficial impact on the cardiovascular system, for instance, via antioxidant, endothelium, and lipid-related influences. For example, in vitro evidence has shown that vitamin C, such as from citrus fruits and bell peppers, can increase nitric oxide bioavailability, which can induce vasodilation and promote the integrity of the endothelium and hence lower blood pressure [49]. Vitamin C may also protect cell constituents against oxidative stress and aid in the synthesis of several relevant biomolecules via acting as an enzymatic cofactor, thus playing a pivotal role in several processes involved in the pathogenesis of CVD [50,51]. These antioxidant properties are notable as reducing oxidative stress is believed to be a key factor in the prevention of cardiovascular pathology [52]. Various vegetables and fruits are also a source of polyphenols, such as flavonoids, phenolic acids, and lignans, which may also have antioxidant action and reduce levels of LDL-C, triglycerides, and so on, thus ameliorating cardiovascular complications [53]. Likewise, carotenoids, found in various brightly colored produce, and their metabolites’ effects have been implicated in cardiovascular health due to intracellular signaling cascades that influence gene expression, reducing LDL-C plasma levels [54] and promoting HDL-C functionality [55], as well as their antioxidant and anti-inflammatory properties. In a systematic review and meta-analysis involving 69 prospective studies, the relationship between dietary intake and/or blood concentrations of vitamin C, carotenoids (total, beta-carotene, alpha-carotene, beta-cryptoxanthin, and lycopene), and alpha-tocopherol, serving as indicators of vegetable and fruit consumption, was associated with a diminished risk of CHD, stroke, CVD, and all-cause mortality [56]. Vegetables and fruits are also sources of potassium, with higher levels found in foods such as potatoes, tomatoes, bananas, and oranges, and increased intake of potassium, in particular higher potassium to sodium ratio, has been linked to lower blood pressure [57]. Higher dietary fiber, such as from broccoli and berries, has also been related to reduced hypertension and better cardiovascular health, possibly because of its beneficial effects on lipid profile and endothelial function [58]. Higher fiber intake can also improve insulin sensitivity, which could play several beneficial effects on other cardiovascular risk factors, decreasing the risk of CVD [58]. Consuming a combination of nutrients and bioactives from a variety of vegetables and fruits may offer synergistic cardiometabolic benefits; however, current evidence is inconclusive. Furthermore, Kegler and colleagues have shown that consuming a greater variety of different vegetables and fruits is associated with a greater likelihood of meeting fruit and vegetable guidelines [41]. This is of importance as globally, 78% of individuals do not meet the World Health Organization (WHO) recommendations for five daily servings of fruits and vegetables [59]. Moreover, poor diets have an economic burden, especially for governments and healthcare costs. Higher fruit and vegetable frequency and variety have been associated with reductions in economic burden, albeit small, particularly in healthcare claims, and lower healthcare costs [60,61].

### 4.2. Limitations and Strengths

Several limitations of the present study should be acknowledged. Firstly, there was evidence of inconsistency in a few of the pooled estimates. All-cause and CHD mortality were downgraded for serious inconsistency due to substantial unexplained heterogeneity. It is noteworthy that the included studies in the present analyses used different criteria to define the exposure. These divergent exposure criteria could affect the accuracy of the risk estimates and describe some of the heterogeneity; however, due to the limited number of studies, subgroup analyses could not be confidently conducted to explore the influence of those definitions on our results. Second, there was evidence of imprecision in most of the pooled analyses. As such, findings were downgraded for serious imprecision owing to the crossing of the prespecified MID, which meant that clinically important beneficial associations could not be ruled out. Furthermore, the number of studies identified and included in each outcome analysis was relatively small. Consequently, future studies are likely to change the pooled risk estimates. Furthermore, subgroup analyses were not able to confidently be performed to explore potential sources of heterogeneity. Likewise, publication bias could not be investigated due to the low number of study comparisons (less than 10). This is notable as a significant level of inter-study heterogeneity was detected in the analyses of a variety of vegetable and fruit consumption and all-cause and CHD mortality. Moreover, the limited number of eligible studies prevented the ability to perform sex-based comparisons, assessing whether the associations varied between men and women or the baseline health status of participants. Despite the intention to conduct dose-response meta-regression analyses, due to the limited available data, these analyses could not be performed with confidence despite the best efforts to contact authors where relevant. This limitation is noteworthy as dose-response meta-regression analyses enable the exploration of potential divergent associations based on different exposure levels, offering a more comprehensive understanding of the associations to guide clinical decisions.

The present analysis has several strengths. It offers a thorough synthesis of the existing published literature concerning the potential variety in the intakes of vegetables and fruits in patient-important and surrogate CVD outcomes. A systematic search strategy was used to capture all pertinent cross-sectional and prospective cohort studies. It is relevant to note that the study design was not restricted in the search strategy, and relevant randomized controlled trials were not identified in the search. Further, the GRADE approach was used to assess the certainty of the evidence.

### 4.3. Practical Implications

Quantity of vegetable and fruit intake is undoubtedly important for cardiovascular and overall health and tends to be underscored in guidelines and clinical practice, whereas *variety* is often overlooked. Yet, emphasis on variety may hold a distinct significance. Findings from the present study of the impact of variety, independent of the amount of vegetables and fruits, on cardiovascular health and mortality have important clinical implications as they may aid in informing the development of future dietary strategies to prevent or delay mortality and provide greater insight in relation to cardiovascular health, in promoting healthy aging.

Furthermore, taking a holistic approach to vegetable and fruit intake, encompassing amount and variety, may confer a more robust protection against chronic diseases, making dietary diversity a key factor to consider in the promotion of optimal health outcomes. Nonetheless, future research is needed to further elucidate the effects of increasing variety, independent of the amount of vegetable and fruit consumption, on cardiovascular risk factors or the prevention of disease.

## 5. Conclusions

Current evidence supports the use of variety as having a beneficial role, independent of amount, in the consumption of vegetables and fruits in relation to curbing all-cause mortality while highlighting the need for additional studies with a higher degree of evidence to better understand and clarify the role of variety (over and above amount) of vegetable and fruit intake in cardiovascular health.

## Figures and Tables

**Table 1:** Summary of the characteristics of the included studies.

Characteristic	Cross-Sectional Studies	Prospective Cohort Studies
Number of reports	5	7
Study location	Iran (1), United States (4)	China (1), Japan (1), the Netherlands (1), Spain (1), the United Kingdom (1), The United States (2)
Sample size (range)	1159 (98 to 38,981)	24,601 (401 to 79,904)
Baseline age (range)	46.8 y (18 to 98 y)	54.7 y (20 to 76 y)
Duration (range)	N/A	12 y (1 to 24 y)
Dietary assessment method	FFQ (2), 24-h recall (1), Food Record (1), Survey (1)	FFQ (6), 24-h recall (1)
Outcome assessment method	Conducted by investigators (3), self-reported (2)	Confirmed or conducted by investigators (7)

Data are presented as median (range) or number of cohorts. FFQ = food frequency questionnaire; N/A = not applicable.

## Data Availability

Data described in the manuscript and analytic code will be made available upon request.

## Supplementary Materials

The following supporting information can be downloaded at: https://www.mdpi.com/article/10.3390/nu15234913/s1, Figure S1: Forest plot of prospective cohort studies of the association between variety of vegetable and/or fruit intake and all-cause mortality; Figure S2: Forest plot of prospective cohort studies of the association between variety of vegetable and/or fruit intake and CVD mortality; Figure S3: Forest plot of prospective cohort studies of the association between variety of vegetable and/or fruit intake and CHD mortality; Figure S4: Forest plot of prospective cohort studies of the association between variety of vegetable and/or fruit intake and CHD incidence; Figure S5: Forest plot of prospective cohort studies of the association between variety of vegetable and/or fruit intake and stroke incidence; Figure S6: Forest plot of cross-sectional cohort studies of the association between variety of vegetable and/or fruit intake and CVD prevalence; Figure S7: Forest plot of cross-sectional studies of the association between variety of vegetable and/or fruit intake and CHD prevalence; Figure S8: Forest plot of cross-sectional studies of the association between variety of vegetable and/or fruit intake and stroke prevalence; Figure S9: Forest plot of prospective cohort studies of the association between variety of vegetable and/or fruit intake and hypercholesterolemia; Figure S10: Forest plot of prospective cohort studies of the association between variety of vegetable and/or fruit intake and high LDL-C; Figure S11: Forest plot of prospective cohort studies of the association between variety of vegetable and/or fruit intake and LDL-C; Figure S12: Forest plot of prospective cohort studies of the association between variety of vegetable and/or fruit intake and low HDL-C; Figure S13: Forest plot of prospective cohort studies of the association between variety of vegetable and/or fruit intake and HDL-C; Figure S14: Forest plot of prospective cohort studies of the association between variety of vegetable and/or fruit intake and hypertriglyceridemia; Figure S15: Forest plot of prospective cohort studies of the association between variety of vegetable and/or fruit intake and triglycerides; Figure S16: Forest plot of cross-sectional cohort studies of the association between variety of vegetable and/or fruit intake and hypertension; Figure S17: Forest plot of prospective cohort studies of the association between variety of vegetable and/or fruit intake and systolic blood pressure; Figure S18: Forest plot of prospective cohort studies of the association between variety of vegetable and/or fruit intake and diastolic blood pressure; Figure S19: Forest plot of cross-sectional studies of the association between variety of vegetable and/or fruit intake and obesity; Figure S20: Forest plot of prospective cohort studies of the association between variety of vegetable and/or fruit intake and body weight; Figure S21: Forest plot of prospective cohort studies of the association between variety of vegetable and/or fruit intake and waist circumference; Figure S22: Forest plot of prospective cohort studies of the association between variety of vegetable and/or fruit intake and C-reactive protein; Figure S23: Forest plot of cross-sectional studies of the association between variety of vegetable and/or fruit intake and diabetes; Figure S24: Forest plot of prospective cohort studies of the association between variety of vegetable and/or fruit intake and fasting glucose; Figure S25: Forest plot of prospective cohort studies of the association between variety of vegetable and/or fruit intake and hemoglobin A1c; Figure S26: Forest plot of prospective cohort studies of the association between variety of vegetable and/or fruit intake and CCMR; Figure S27: Forest plot of prospective cohort studies of the association between variety of vegetable and/or fruit intake and Framingham Risk Score; Figure S28: Sensitivity analysis of the systematic removal of each cohort for the association between variety of vegetable and/or fruit intake and all-cause mortality in prospective cohorts; Figure S29: Sensitivity analysis of the systematic removal of each cohort for the association between variety of vegetable and/or fruit intake and cardiovascular mortality in prospective cohorts; Figure S30: Sensitivity analysis of the systematic removal of each cohort for the association between variety of vegetable and/or fruit intake and CHD incidence in prospective cohorts; Figure S31: Sensitivity analysis of the systematic removal of each cohort for the association between variety of vegetable and/or fruit intake and systolic blood pressure in prospective studies; Figure S32: Sensitivity analysis of the systematic removal of each cohort for the association between variety of vegetable and/or fruit intake and HDL-C in prospective cohorts; Figure S33: Sensitivity analysis of the systematic removal of each cohort for the association between variety of vegetable and/or fruit intake and triglycerides in prospective cohorts; Figure S34: Sensitivity analysis of the systematic removal of each cohort for the association between variety of vegetable and/or fruit intake and waist circumference in prospective cohorts; Figure S35: Sensitivity analysis of the systematic removal of each cohort for the association between variety of vegetable and/or fruit intake and diabetes in cross-sectional studies; Table S1: PRISMA 2020 Checklist; Table S2: MOOSE Checklist; Table S3: PECOTS framework of the search strategy; Table S4: Search strategy; Table S5: Characteristics of included cross-sectional studies; Table S6: Characteristics of included prospective cohort studies; Table S7: Variety of vegetable and fruit intake assessment methods; Table S8: Confounding variables of included cross-sectional studies; Table S9: Confounding variables of included prospective studies; Table S10: Risk of bias scores of included observational cohort studies; Table S10a: Study Quality Assessment Tool for Observational Cohort and Cross-sectional Studies from the National Heart, Lung, and Blood Institute scores of included cross-ectional studies; Table S10b: Newcastle-Ottawa Scale (NOS) scores of included prospective cohort studies; Table S11: GRADE assessment.

## Abbreviations

BMI, body mass index; CI, confidence interval; CVD, cardiovascular disease; FFQ, food frequency questionnaire; HR, hazard ratios; MOOSE, Meta-analysis of Observational Studies in Epidemiology; OR, odds ratio; PECOTS, population, exposure, comparator, outcome, timeline, setting/study design framework; RR, risk ratio.
