# Supplementary material for: Vegetable and Fruit Intake Variety and Cardiovascular Health and Mortality: A Systematic Review and Meta-Analysis of Observational Studies
Source: Nutrients. 2023 Nov 24;15(23):4913. doi: 10.3390/nu15234913 (PMC10707746; doi:10.3390/nu15234913)
Supplement: Supplementary file 1 [file nutrients-15-04913-s001.zip › nutrients-2685765-supplementary.pdf]

Online Supplemental Material

Re: Nishi et al. Variety of Vegetable and Fruit Intake in Association with Cardiovascular Health and All-Cause Mortality: A Systematic Review and Meta-Analysis of Observational Studies

Table of Contents

Supplemental Tables **4**

Supplemental Table S1: PRISMA 2020 Checklist 4

Supplemental Table S2: MOOSE (Meta-analyses of Observational Studies in Epidemiology) Checklist 7

Supplemental Table S3: PECOTS framework of the search strategy 9

Supplemental Table S4: Search strategy 10

Supplemental Table S5. Characteristics of included cross-sectional studies. 11

Supplemental Table S6. Characteristics of included prospective cohort studies 13

Supplemental Table S7. Variety of vegetable and fruit intake assessment methods. 15

Supplemental Table S8. Confounding variables of included cross-sectional studies 22

Supplemental Table S9. Confounding variables of included prospective cohort studies. 23

Supplemental Table S10. Risk of bias scores of included observational cohort studies 25

Supplemental Table S10a. Study Quality Assessment Tool for Observational Cohort and Cross-sectional Studies from the National Heart, Lung, and Blood Institute scores of included cross-sectional studies 25

Supplemental Table S10b. Newcastle-Ottawa Scale (NOS) scores of included prospective cohort studies 26

Supplemental Table S11. GRADE assessment 27

Supplementary Figures **31**

Supplemental Figure S1. Forest plot of prospective cohort studies of the association between variety of vegetable and/or fruit intake and all-cause mortality. 31

Supplemental Figure S2. Forest plot of prospective cohort studies of the association between variety of vegetable and/or fruit intake and CVD mortality. 31

Supplemental Figure S3. Forest plot of prospective cohort studies of the association between variety of vegetable and/or fruit intake and CHD mortality. 32

Supplemental Figure S4. Forest plot of prospective cohort studies of the association between variety of vegetable and/or fruit intake and CHD incidence. 32

Supplemental Figure S5. Forest plot of prospective cohort studies of the association between variety of vegetable and/or fruit intake and stroke incidence. 33

Supplemental Figure S6. Forest plot of cross-sectional cohort studies of the association between variety of vegetable and/or fruit intake and CVD prevalence. 33

Supplemental Figure S7. Forest plot of cross-sectional cohort studies of the association between variety of vegetable and/or fruit intake and CHD prevalence. 34
Supplemental Figure S8. Forest plot of cross-sectional cohort studies of the association between variety of vegetable and/or fruit intake and stroke prevalence. 34
Supplemental Figure S9. Forest plot of prospective cohort studies of the association between variety of vegetable and/or fruit intake and hypercholesterolemia. 35
Supplemental Figure S10. Forest plot of prospective cohort studies of the association between variety of vegetable and/or fruit intake and high LDL-C. 35
Supplemental Figure S11. Forest plot of prospective cohort studies of the association between variety of vegetable and/or fruit intake and LDL-C. 36
Supplemental Figure S12. Forest plot of prospective cohort studies of the association between variety of vegetable and/or fruit intake and low HDL-C. 36
Supplemental Figure S13. Forest plot of prospective cohort studies of the association between variety of vegetable and/or fruit intake and HDL-C. 37
Supplemental Figure S14. Forest plot of prospective cohort studies of the association between variety of vegetable and/or fruit intake and hypertriglyceridemia. 37
Supplemental Figure S15. Forest plot of prospective cohort studies of the association between variety of vegetable and/or fruit intake and triglycerides. 38
Supplemental Figure S16. Forest plot of cross-sectional cohort studies of the association between variety of vegetable and/or fruit intake and hypertension. 38
Supplemental Figure S17. Forest plot of prospective cohort studies of the association between variety of vegetable and/or fruit intake and systolic blood pressure. 39
Supplemental Figure S18. Forest plot of prospective cohort studies of the association between variety of vegetable and/or fruit intake and diastolic blood pressure. 39
Supplemental Figure S19. Forest plot of cross-sectional studies of the association between variety of vegetable and/or fruit intake and obesity 40
Supplemental Figure S20. Forest plot of prospective cohort studies of the association between variety of vegetable and/or fruit intake and body weight. 40
Supplemental Figure S21. Forest plot of prospective cohort studies of the association between variety of vegetable and/or fruit intake and waist circumference. 41
Supplemental Figure S22. Forest plot of prospective cohort studies of the association between variety of vegetable and/or fruit intake and C-reactive protein. 41
Supplemental Figure S23. Forest plot of cross-sectional studies of the association between variety of vegetable and/or fruit intake and diabetes. 42
Supplemental Figure S24. Forest plot of prospective cohort studies of the association between variety of vegetable and/or fruit intake and fasting glucose. 42
Supplemental Figure S25. Forest plot of prospective cohort studies of the association between variety of vegetable and/or fruit intake and hemoglobin A1c. 43
Supplemental Figure S26. Forest plot of prospective cohort studies of the association between variety of vegetable and/or fruit intake and CCMR 43

Supplemental Figure S27. Forest plot of prospective cohort studies of the association between variety of vegetable and/or fruit intake and Framingham Risk Score. 44
Supplemental Figure S28. Sensitivity analysis of the systematic removal of each cohort for the association between variety of vegetable and/or fruit intake and all-cause mortality in prospective cohorts. 44
Supplemental Figure S29. Sensitivity analysis of the systematic removal of each cohort for the association between variety of vegetable and/or fruit intake and cardiovascular mortality in prospective cohorts. 45
Supplemental Figure S30. Sensitivity analysis of the systematic removal of each cohort for the association between variety of vegetable and/or fruit intake and coronary heart disease incidence in prospective cohorts. 45
Supplemental Figure S31. Sensitivity analysis of the systematic removal of each cohort for the association between variety of vegetable and/or fruit intake and systolic blood pressure in prospective cohorts. 46
Supplemental Figure S32. Sensitivity analysis of the systematic removal of each cohort for the association between variety of vegetable and/or fruit intake and HDL-C in prospective cohorts. 46
Supplemental Figure S33. Sensitivity analysis of the systematic removal of each cohort for the association between variety of vegetable and/or fruit intake and triglycerides in prospective cohorts. 47
Supplemental Figure S34. Sensitivity analysis of the systematic removal of each cohort for the association between variety of vegetable and/or fruit intake and waist circumference in prospective cohorts. 47
Supplemental Figure S35. Sensitivity analysis of the systematic removal of each cohort for the association between variety of vegetable and/or fruit intake and diabetes in cross-sectional studies. 48
References 49

Supplemental Tables

Supplemental Table S1. PRISMA 2020 Checklist.

Section and Topic	Item #	Checklist item	Location where item is reported
**TITLE**			
Title	1	Identify the report as a systematic review.	1
**ABSTRACT**			
Abstract	2	See the PRISMA 2020 for Abstracts checklist.	3
**INTRODUCTION**			
Rationale	3	Describe the rationale for the review in the context of existing knowledge.	5-6
Objectives	4	Provide an explicit statement of the objective(s) or question(s) the review addresses.	6
**METHODS**			
Eligibility criteria	5	Specify the inclusion and exclusion criteria for the review and how studies were grouped for the syntheses.	6-7, Supplemental Table S3
Information sources	6	Specify all databases, registers, websites, organisations, reference lists and other sources searched or consulted to identify studies. Specify the date when each source was last searched or consulted.	7-8
Search strategy	7	Present the full search strategies for all databases, registers and websites, including any filters and limits used.	Supplemental Table S4
Selection process	8	Specify the methods used to decide whether a study met the inclusion criteria of the review, including how many reviewers screened each record and each report retrieved, whether they worked independently, and if applicable, details of automation tools used in the process.	Figure 1
Data collection process	9	Specify the methods used to collect data from reports, including how many reviewers collected data from each report, whether they worked independently, any processes for obtaining or confirming data from study investigators, and if applicable, details of automation tools used in the process.	7-8
Data items	10a	List and define all outcomes for which data were sought. Specify whether all results that were compatible with each outcome domain in each study were sought (e.g. for all measures, time points, analyses), and if not, the methods used to decide which results to collect.	8-9
	10b	List and define all other variables for which data were sought (e.g. participant and intervention characteristics, funding sources). Describe any assumptions made about any missing or unclear information.	7-9, Supplemental Tables 5-6
Study risk of bias assessment	11	Specify the methods used to assess risk of bias in the included studies, including details of the tool(s) used, how many reviewers assessed each study and whether they worked independently, and if applicable, details of automation tools used in the process.	9-10
Effect measures	12	Specify for each outcome the effect measure(s) (e.g. risk ratio, mean difference) used in the synthesis or presentation of results.	10-11
Synthesis methods	13a	Describe the processes used to decide which studies were eligible for each synthesis (e.g. tabulating the study intervention characteristics and comparing against the planned groups for each synthesis (item #5)).	8-11, Supplemental Tables 5-6
	13b	Describe any methods required to prepare the data for presentation or synthesis, such as handling of missing summary statistics, or data conversions.	10-11

Section and Topic	Item #	Checklist item	Location where item is reported
	13c	Describe any methods used to tabulate or visually display results of individual studies and syntheses.	8, Supplemental Tables 5-6
	13d	Describe any methods used to synthesize results and provide a rationale for the choice(s). If meta-analysis was performed, describe the model(s), method(s) to identify the presence and extent of statistical heterogeneity, and software package(s) used.	10-11
	13e	Describe any methods used to explore possible causes of heterogeneity among study results (e.g. subgroup analysis, meta-regression).	10-11
	13f	Describe any sensitivity analyses conducted to assess robustness of the synthesized results.	10-11
Reporting bias assessment	14	Describe any methods used to assess risk of bias due to missing results in a synthesis (arising from reporting biases).	9-10
Certainty assessment	15	Describe any methods used to assess certainty (or confidence) in the body of evidence for an outcome.	11
**RESULTS**			
Study selection	16a	Describe the results of the search and selection process, from the number of records identified in the search to the number of studies included in the review, ideally using a flow diagram.	Figure 1
	16b	Cite studies that might appear to meet the inclusion criteria, but which were excluded, and explain why they were excluded.	11-13
Study characteristics	17	Cite each included study and present its characteristics.	Supplemental Tables 5-6
Risk of bias in studies	18	Present assessments of risk of bias for each included study.	Supplemental Table S10
Results of individual studies	19	For all outcomes, present, for each study: (a) summary statistics for each group (where appropriate) and (b) an effect estimate and its precision (e.g. confidence/credible interval), ideally using structured tables or plots.	Figures 2-4, Supplemental Figures 1-27
Results of syntheses	20a	For each synthesis, briefly summarise the characteristics and risk of bias among contributing studies.	12-14
	20b	Present results of all statistical syntheses conducted. If meta-analysis was done, present for each the summary estimate and its precision (e.g. confidence/credible interval) and measures of statistical heterogeneity. If comparing groups, describe the direction of the effect.	13-20, Figures 2-4, Supplemental Figures 1-27
	20c	Present results of all investigations of possible causes of heterogeneity among study results.	15-21, Supplemental Figures 28-47
	20d	Present results of all sensitivity analyses conducted to assess the robustness of the synthesized results.	20-21, Supplemental Figures 28-47
Reporting biases	21	Present assessments of risk of bias due to missing results (arising from reporting biases) for each synthesis assessed.	14-15
Certainty of evidence	22	Present assessments of certainty (or confidence) in the body of evidence for each outcome assessed.	15-21, Supplemental Table S11
**DISCUSSION**			

Section and Topic	Item #	Checklist item	Location where item is reported
Discussion	23a	Provide a general interpretation of the results in the context of other evidence.	21-25
	23b	Discuss any limitations of the evidence included in the review.	24-25
	23c	Discuss any limitations of the review processes used.	24-25
	23d	Discuss implications of the results for practice, policy, and future research.	22-25
**OTHER INFORMATION**			
Registration and protocol	24a	Provide registration information for the review, including register name and registration number, or state that the review was not registered.	Abstract, 6
	24b	Indicate where the review protocol can be accessed, or state that a protocol was not prepared.	Abstract, 6
	24c	Describe and explain any amendments to information provided at registration or in the protocol.	NA
Support	25	Describe sources of financial or non-financial support for the review, and the role of the funders or sponsors in the review.	26
Competing interests	26	Declare any competing interests of review authors.	26
Availability of data, code and other materials	27	Report which of the following are publicly available and where they can be found: template data collection forms; data extracted from included studies; data used for all analyses; analytic code; any other materials used in the review.	27

From: Page MJ, McKenzie JE, Bossuyt PM, Boutron I, Hoffmann TC, Mulrow CD, et al. The PRISMA 2020 statement: an updated guideline for reporting systematic reviews. BMJ 2021;372:n71. doi: 10.1136/bmj.n71.

Supplemental Table S2. MOOSE (Meta-analyses of Observational Studies in Epidemiology) Checklist[a].

Reporting Criteria	Reported (Yes/No)	Reported on Page No.
Reporting of Background		
Problem definition	Yes	3, 5-6
Description of Study Outcome(s)	Yes	6-9
Type of exposure or intervention used	Yes	6-7
Study population	Yes	6-7
Reporting of Search Strategy		
Qualifications of searchers (eg, librarians and investigators)	Yes	1
Search strategy, including time period included in the synthesis and keywords	Yes	6-7, Supplemental Tables 3-4
Effort to include all available studies, including contact with authors	Yes	11
Databases and registries searched	Yes	7
Search software used, name and version, including special features used (eg, explosion)	Yes	8
Use of hand searching (eg, reference lists of obtained articles)	Yes	7, Figure 1, Supplemental Table S3
List of citations located and those excluded, including justification	Yes	Figure 1
Method for addressing articles published in languages other than English	Yes	7
Method of handling abstracts and unpublished studies	Yes	7, Supplemental Tables 3-4
Description of any contact with authors	Yes	8, 11
Reporting of Methods		
Description of relevance or appropriateness of studies assembled for assessing the hypothesis to be tested	Yes	7
Rationale for the selection and coding of data (eg., sound clinical principles or convenience)	Yes	7,8
Documentation of how data were classified and coded (eg., multiple raters, blinding, and interrater reliability)	Yes	7,8
Assessment of confounding (eg., comparability of cases and controls in studies were appropriate)	Yes	7-8
Reporting Criteria		
Assessment of study quality, including blinding of quality assessors; stratification or regression on possible predictors of study results	Yes	9

Assessment of heterogeneity	Yes	10
Description of statistical methods (eg., complete description of fixed or random effects models, justification of whether the chosen models account for predictors of study results, dose-response models, or cumulative meta-analysis) in sufficient detail to be replicated	Yes	9-11
Provision of appropriate tables and graphics	Yes	Table 1, Figures 1-4, Supplemental Material
Reporting of Results		
Table giving descriptive information for each study included	Yes	Table 1, Supplemental Table S5
Results of sensitivity testing (eg., subgroup analysis)	Yes	20-21, Supplemental Figures 9-27
Indication of statistical uncertainty of findings	Yes	21, Supplemental Table S11
Reporting of Discussion		
Quantitative assessment of bias (eg., publication bias)	Yes	21
Justification for exclusion (eg., exclusion of non-English-language citations)	Yes	NA
Assessment of quality of included studies	Yes	8, Supplemental Tables 10a-b
Reporting of Conclusions		
Consideration of alternative explanations for observed results	Yes	21-24
Generalization of the conclusions (ie, appropriate for the data presented and within the domain of the literature review)	Yes	21-24
Guidelines for future research	Yes	24
Disclosure of funding source	Yes	26

NA = Not applicable. [a]Stroup DF, Berlin JA, Morton SC, Olkin I, Williamson GD, Rennie D, Moher D, Becker BJ, Sipe TA, Thacker SB. Meta-analysis of observational studies in epidemiology: a proposal for reporting. Meta-analysis Of Observational Studies in Epidemiology (MOOSE) group. JAMA. 2000 Apr 19;283(15):2008-12. doi: 10.1001/jama.283.15.2008. PMID: 10789670.

Supplemental Table S3. PECOTS framework of the search strategy.

PECOTS framework[a] defined in the present systematic review and meta-analysis					
**Participants**	**Exposure**	**Comparators**	**Outcomes**	**Time/ Duration**	**Setting/ Study Design**
Adults (≥ 18 years) of any sex, gender, and ethnicity, and free of CVD at baseline (for analysis of CVD-related incidence) otherwise of any health status.	Higher variety of vegetable and/or fruit consumption in the diet.	Lower variety of vegetable and/or fruit consumption in the diet.	*CVD risk factors:*<br>LDL-C<br>HDL-C<br>TG<br>TC<br>SBP<br>DBP<br>WC<br>Body weight<br>Inflammation<br>Fasting blood glucose<br>Risk scores<br><br>*CVD prevalence:*<br>Overall CVD<br>CHD<br>Stroke<br><br>*CVD incidence:*<br>Overall CVD<br>CHD<br>Stroke<br><br>*Mortality:*<br>CVD-related<br>CHD-related<br>Stroke-related<br>All-cause	Cross-sectional (one time point)<br>or<br>prospective cohort (at least 1 year in duration).<br><br>Setting being the general population.	

Abbreviations: CHD, coronary heart disease; CVD, cardiovascular disease; LDL-C, low-density lipoprotein-cholesterol; HDL-C, high-density lipoprotein-cholesterol; TG, triglycerides; TC, total cholesterol; SBP, systolic blood pressure; DBP, diastolic blood pressure; WC, waist circumference.
[a]Moher D, Shamseer L, Clarke M, Ghersi D, Liberati A, Petticrew M, Shekelle P, Stewart LA and PRISMA-P Group. Preferred reporting items for systematic review and meta-analysis protocols (PRISMA-P) 2015 statement. Systematic Reviews 2015; 4:1. https://doi.org/10.1186/2046-4053-4-1.

Supplemental Table S4. Search strategy.

Database	Search Period	Search Terms
MEDLINE-PubMed	1946 to Mar. 21st, 2023	(((fruit[MeSH Terms]) OR (fruit*) OR (vegetable*) OR (vegetable[MeSH Terms])) AND (variety) AND ((coronary disease*) OR (stroke) OR (cardiovascular diseases[MeSH Terms]) OR (cardiovascular disease) OR (cholesterol) OR (cholesterol[MeSH Terms]) OR (triglycerides) OR (blood pressure) OR (body weight) OR (waist circumference) OR (body mass index) OR (blood glucose) OR (insulin resistance) OR (hemoglobin 1ac) OR (cardiovascular risk*) OR (cardiovascular morbidity) OR (cardiovascular mortality)))
The Cochrane Library	1946 to Mar. 21st, 2023	#1 MeSH descriptor: [Fruit] explode all trees<br>#2 fruit*<br>#3 vegetable*<br>#4 MeSH descriptor: [Vegetables] explode all trees<br>#5 #1 OR #2 OR #3 OR #4<br>#6 variety<br>#7 coronary disease*<br>#8 stroke<br>#9 MeSH descriptor: [Cardiovascular Diseases] explode all trees<br>#10 cardiovascular disease<br>#11 cholesterol<br>#12 triglycerides<br>#13 blood pressure<br>#14 body weight<br>#15 waist circumference<br>#16 body mass index<br>#17 blood glucose<br>#18 insulin resistance<br>#19 hemoglobin A1c<br>#20 cardiovascular risk*<br>#21 cardiovascular mortality<br>#22 cardiovascular morbidity<br>#23 #7 OR #8 OR #9 OR #10 OR #11 OR #12 OR #13 OR #14 OR #15 OR #16 OR #17 OR #18 OR #19 OR #20 OR #21 OR #22<br>#24 #5 AND #6 AND #23

Abbreviations: MeSH, Medical Subject Headings.

Supplemental Table S5. Characteristics of included cross-sectional studies.

First Author, Journal, Year	Study Design	Cohort	Country	Data Collection Years	Baseline Health Status	N	Age, y	Sex (%W)	Relevant Outcomes	Outcome assessment method	Diet assessment method	Exposure	Funding source
Azadbakht et al., Pub Health Nutr, 2006	CS	TLGS	Iran	Implemented: 1999	No noted restrictions	581	38.6 (>18)	49.2%	Obesity: (BMI ≥30 kg/m2) Hypercholesterolemia: (TC≥6.2 mmol/L) Low HDL-C: (<1.03 mmol/L) Hypertriglyceridemia: (TG ≥2.3 mmol/L) Diabetes: (FGB≥7.0 mmol/L or 2HPG≥11.1 mmol/L) Hypertension: (SBP ≥140 mm Hg or DBP ≥90 mm Hg) High LDL-C: (≥4.1 mmol/L)	Height, weight, and blood pressure measured, fasting blood samples collected, 75g oral glucose tolerance test.	Validated, semi-quantitative FFQ. Trained-dietitian interview.	Vegetable variety, fruit variety	Agency
Bernstein et al., J Am Diet Assoc, 2002	CS	FICSIT	USA	N/A (Prior to 1993)	Frail nursing home residents	98	87.1±0.6 (72-98)	63%	BMI, HDL-C, VLDL[a] TG[a]	Height and weight were measured, blood samples collected.	3-day weighted food records of 3 consecutive days of the week	Fruit and vegetables variety	Agency - Industry
Bhupathiraju et al., AJCN, 2011	CS	BPRHS	USA	Recruitment: 2000	No noted restrictions	1159	56.9 (45-75)	63%	FRS, CRP	Height and weight were measured, blood samples collected.	Validated semiquantitative FFQ	Vegetable and fruit variety	None reported
Conrad et al., Nutr J, 2018	CS	NHANES 1999-2014	USA	1999 to 2014	No noted restrictions	38981	~46.8 (>20)	52%	CVD incidence, CHD incidence, Stroke incidence, Diabetes incidence	Self-reported	24-h dietary recalls by trained interviewers	Vegetable variety	Agency
Kegler et al., BMC Public Health, 2021	CS	N/A	USA	2015	No noted restrictions	4942	44.4±15.4 (18-75)	51.7%	Overweight/Obesity	Self-reported	Online survey	Vegetable and fruit variety	Agency

Abbreviations: BPRHS = Boston Puerto Rican Health Study, BMI = body mass index, CHD = coronary heart disease, CRP = C-reactive protein, CS = cross-sectional, CVD = cardiovascular disease, DBP = diastolic blood pressure, FBG = fasting blood glucose, FFQ = food frequency questionnaire, FICSIT = Frailty and Injuries: Cooperative Studies of Intervention Techniques, FRS = Framingham Risk Score, HDL-C = high-density lipoprotein-cholesterol, HPFS = Health

Professionals Follow-up Study, N/A = not available, NHANES = National Health and Nutrition Examination Survey, NHS = Nurses Health Study, TC = total cholesterol, TG = triglycerides, TLGS = Tehran Lipid and Glucose Study, USA = United States of America, W = women.
[a]data presented as log transformed beta-coefficients and hence could not be appropriately included in the current analyses [1–4].

Supplemental Table S6. Characteristics of included prospective cohort studies.

First Author, Journal, Year	Study Design	Cohort	Country	Data Collection Years	Duration, y	Baseline Health Status	N	Age, y	Sex (% W)	Relevant Outcomes	Outcome assessment method	Diet assessment method	Exposure	Funding source
Bhupathiraju et al., Am J Clin Nutr, 2013	P	NHS & HPFS	USA	NHS: 1984 to 2008 HPFS: 1986 to 2008	NHS: 24 years (1984 to 2008); HPFS: 22 years (1986 to 2008)	Free of cancer, diabetes, and CVD	NHS: 71141 HPFS: 42135	NHS: 50.2 (30 to 55) HPFS: 53.1 (40 to 75)	NHS: 100% HPFS: 0%	CHD mortality, CHD incidence	Medical record, autopsy report, death certificate, US postal system, state vital statistics departments, National Death Index.	Validated, semi-quantitative 126-item FFQ	Vegetable and fruit variety	None reported
Conrad et al., Nutrients, 2018	P	NHANES	USA	NHANES: 1999 to 2010 Linked Mortality Files: 1999 to 2011	6.5 years (mean) (1999 to 2011)	No noted restrictions	29133	46.3 (95% CI: 45.8 to 46.7)	52.2%	All-cause mortality, CVD mortality, CHD mortality	Dietary data and mortality data were linked using different institutional database: WWEIA; NCHS; NDI	24-h dietary recalls by trained interviewers	Vegetable variety	Agency
Kobayashi et al., Eur J Clin Nutr, 2019	P	JPHC study	Japan	1995 to 2012	14.9 years (median) (1995 to 2012)	Free of CVD and cancer	79904	56.3 (45-74)	53.4%	All-cause mortality, CVD mortality	Death certificates. Residencial registry to confirm residence status	Self-administered FFQ	Vegetable variety, fruits variety	Agency
Lamb et al., Eur J Clin Nutr, 2017	P	ADDITION-Cambridge study	UK	Recruitment: 2000 to 2006	5 years (Recruitment: 2001-2003)	Free of disease, but with high diabetes risk	401	61.4 (40-69)	43.4	SBP, HDL-C, TG, WC, HbA1c, CCMR	Anthropometric and blood pressure outcomes were measured. Blood samples collected and analyzed. CCMR calculated including: WC, blood pressure, HbA1c, TG and HDL-C.	Validated 130-item FFQ. Self-administered.	Vegetable and fruit variety	Agency

López-González et al., Eur J Clin Nutr, 2022	P	PREDIMED-Plus	Spain	Recruitment: 2013 to 2016	1 year	MetS and OW/OB	6647	65.0 (Women: 60-75; Men: 55-75)	48.4%	SBP, DBP, LDL-C, HDL-C, TG, FBG, BMI, BW, WC	Anthropometric and blood pressure outcomes were measured. Blood samples collected and analyzed. LDL-C: was calculated using the Friedewald formula.	Validated, semi-quantitative 143-item FFQ. Administered by trained dietitians.	Vegetable and fruit variety	Agency
Oude Griep et al., Pub Health Nutr, 2012	P	MORGEN Study	Netherlands	Baseline: 1993 to 1997	10 years (Baseline 1993-1997 to 2006)	Free of CVD and diabetes	20069	41.5 (20-65)	55.2%	CHD incidence, Stroke incidence	Information was obtained from the municipal population register and the hospital discharge register (Fatal CVD: ICD-10 codes 120-125; Fatal stroke: ICD-10 codes 160-167, & 169; non-fatal stroke including transient ischaemic attack: ICD-10 codes 430-438).	Validated, semi-quantitative 178-item FFQ. Self-administered.	Vegetable and fruit variety	Agency
Yeung et al., J Nutr Health Aging, 2021	P	Mr. OS and Ms. OS (Hong Kong) study	China	Baseline: 2001 to 2003	14-years (Recruitment 2001-2003 to 2015-2017)	Free of sarcopenia and/or frailty	3992	72* (68-76)	49.9%	All-cause mortality, CVD mortality	Hong Kong Government Death Registry	Validated, semi-quantitative 280-item FFQ. Administered by trained research staff	Vegetable variety, Fruit variety	Agency

Abbreviations: ADDITION = Anglo–Danish–Dutch Study of Intensive Treatment In People with Screen-Detected Diabetes in Primary Care-Cambridge study, BMI = body mass index, CHD = coronary heart disease, CVD = cardiovascular disease, DBP = diastolic blood pressure, FBG = fasting blood glucose, FFQ = food frequency questionnaire, FRS = Framingham Risk Score, HDL-C = high-density lipoprotein-cholesterol, HPFS = Health Professionals Follow-up Study, JPHC = Japan public health center study, LDL-C = low-density lipoprotein-cholesterol, MetS = metabolic syndrome, MORGEN = The Monitoring Project on Risk Factors and Chronic Diseases in the Netherlands Study, NHANES = National Health and Nutrition Examination Survey, NHS = Nurses Health Study, OB = obesity, OW = overweight, P = prospective cohort; PREDIMED-Plus = PREvención con DIeta MEDiterránea (Prevention with Mediterranean Diet)-Plus, SBP = systolic blood pressure, TG = triglycerides, UK = United Kingdom, USA = United States of America, VLDL = very low-density lipoprotein, W = women, WC = waist circumference [5–11].

Supplemental Table S7. Variety of vegetable and fruit intake assessment methods.

Study	Variety Score Method	Scoring Categories	Primary Components	Cut-offs
Azadbakht et al. 2006	*Dietary intake measurement tool:* 168-item semi-quantitative FFQ<br><br>*Variety score:* Two subgroups of fruits were considered (fruit and fruit juice, berries, and citrus) and vegetables were divided into seven subgroups (vegetables, potatoes, tomatoes, starchy vegetables, legumes, yellow vegetables, green vegetables). To be counted as a 'consumer' for any of the food group categories, a respondent had to consume at least one-half serving, as defined by the Food Guide Pyramid quantity criteria, for one day. It did not need to be eaten all at once. Within the food groups, each of the five broad food categories received a maximum diversity score of 2 out of the 10 possible score points. For calculation of the score of each group, we divided the number of subgroups consumed by the total number of subgroups in each main group and then we multiplied this by two. Within each of the food groups, the score reflects the percentage of the possible maximum score.	Quartiles	Vegetables included: potatoes, tomatoes, starchy vegetables, legumes, yellow vegetables, green vegetables.<br><br>Fruits were not specified in detail.	Q1: <0.8,<br>Q2: 0.8 to <1.3<br>Q3: 1.3 to <1.6<br>Q4: ≥1.6
Berstein et al., 2002	*Dietary intake measurement tool:* 3-day weighed food records on 3 consecutive days of the week.<br><br>*Variety score:* Fruit and vegetable variety was based on the number of different fruits and vegetables consumed in the 3 days, regardless of quantity. Foods that were consumed on multiple occasions during the 3 days were counted only once.<br><br>Variety score was coded manually, directly from the original food records. Foods were counted as they were listed on the food record.<br><br>A food-based approach was used; therefore, items were counted as the whole foods, and foods were not broken down into their contributors--or individual ingredients, which were also listed on the food record for nutrient analysis purposes.	Beta coefficient	Not specified.	Not applicable

Bhupathiraju & Tucker, 2011	*Dietary intake measurement tool:* Semi-quantitative FFQ.<br><br>*Variety score:* Variety in fruit and vegetable intake was defined as the total number of unique fruits and vegetables consumed at least once per month over the past 12 months.	Tertiles	Apples, pears, bananas, oranges, grapefruit, peaches, apricots, nectarine, plums, grapes, avocado, kiwi fruit, papaya, mangoes, prunes, cantaloupe, honeydew melon, watermelon, cherries, strawberries, blueberries, raspberries, cranberries, pineapple, olives, and 100% beets, asparagus, mushrooms, eggplant, onion, squash, cucumber, radish, celery, cilantro, garlic, parsley, zucchini, basil, and 100% vegetable juice. Starchy vegetables (including potato, plantains, tannier, and cassava), beans, and legumes (including lima beans, pinto beans, white beans, black beans, pink beans, kidney beans, fruit juice. Vegetables included lettuce, spinach, tomato, carrots, string beans, peas, corn, peppers, broccoli, cauliflower, cabbage, cowpeas, soybeans, split peas, and lentils) were excluded from the analyses.	Tertile 1: VF intake =17.8 (2.0-22.4)<br>Tertile 2: 26.2 (22.4-29.7)<br>Tertile 3: 34.0 (29.7-44.7)<br><br>Median number of unique vegetables and fruits consumed at least once per month, ranges in parentheses.
Bhupathiraju et al., 2013	*Dietary intake measurement tool:* 126-item semi-quantitative FFQ.<br><br>*Variety score:* Variety in fruit and vegetable intake was defined as the total number of unique fruits and vegetables consumed at least once per week. To account for minor differences in the number of fruit and vegetable items assessed at each follow-up cycle, the variety score was standardized to 30 (11 for fruit score and 19 for vegetable score).	Quintiles	Citrus fruit, green leafy vegetables, cruciferous vegetables, and fruit and vegetables rich in b-carotene, lutein, lycopene, or vitamin C, potatoes, soy, or other legumes	Women (n=1984)<br>Q1: 5.3 ±2.8<br>Q2: 9.0±3.1<br>Q3: 11.4±3.4<br>Q4: 13.5±3.6<br>Q5: 16.4±4.0<br><br>Men (n=1986)<br>Q1: 4.7±2.7<br>Q2: 8.3±3.0<br>Q3: 10.6±3.3<br>Q4: 12.9±3.6<br>Q5: 16.1±4.2

Conrad et al., 2018 (Nutr J)	*Dietary intake measurement tool:* 24-h recall administered by a trained interviewer using United States Department of Agriculture's (USDA) Automated Multiple Pass Method.<br><br>*Variety score*:<br>The Healthy Food Diversity index.<br><br>Intake data from day 1 only was used because this represents group-level intake. The index used to measure vegetable variety was based on the Healthy Food Diversity index, which measures total dietary diversity independent of amount, and penalizes consumption of foods that are discordant with user-defined consumption targets. To measure vegetable variety, we modified the index to focus exclusively on consumption of vegetables, and we used consumption targets that reflect the DGA 2015–2020 vegetable subgroup recommendations (for 2200 kcal/day) for dark green vegetables, red and orange vegetables, legumes, starchy vegetables, and other vegetables. The consumption targets of DGA 2015–2020 are in weekly units (i.e., cup-equivalents per week), so these were converted to daily units (recommended weekly consumption divided by seven) to be consistent with how intake data from WWEIA are measured.<br>The equation for the index is comprised of two parts. The first part is the Berry Index, which measures the number and proportionality of vegetable subgroups reported consumed by an individual. Values are bounded by 0 and 0.8, where the minimum score represents zero vegetable intake, and the maximum score represents equal proportions of all vegetable subgroups. Finally, the Healthy Food Diversity index is computed by multiplying the Berry Index by the Health Value, which ensures that higher index scores are achieved by: 1) consumption of more vegetable subgroups, and 2) greater relative consumption of vegetable subgroups that have greater weighting. The vegetable variety score, using the Healthy Food Diversity index, is bounded by 0 and 0.64.	Quintiles	Not specified	Median vegetable variety scores for each quintile are:<br>Q1: 0,<br>Q2: 0.17,<br>Q3: 0.33,<br>Q4: 0.43,<br>Q5: 0.52.

Conrad et al., 2018 (Nutrients)	*Dietary intake measurement tool:* Trained interviewers administer 24-h dietary recalls to study participants using United States Department of Agriculture's (USDA) Automated Multiple Pass Method.<br><br>*Variety score:* Vegetable variety was measured using an index developed to measure adherence to the DGA-2015 recommendations for daily vegetable variety. The index measures the variety of vegetable intake independent of amount, and penalizes the consumption of vegetable subgroups (dark green vegetables, red and orange vegetables, legumes, starchy vegetables, and other vegetables) that do not align with recommended intake proportions in the DGA 2015–2020. The index contains two parts.<br>1) The Berry Index, assesses the proportionality of vegetable subgroups that individuals report consumed. The consumption targets of DGA 2015–2020 are in weekly units (i.e., cup-equivalents per week), so to be consistent with how WWEIA data are measured, these were converted to daily units. The minimum score (0) represents zero vegetable intake, and the maximum score (0.8) represents equal proportions of all vegetable subgroups.<br>2) The Health Value, gives greater weighting to vegetable subgroups that are recommended in greater proportions. The minimum score (0) represents zero vegetable intake, and the maximum score (1) represents the consumption of only the subgroups with the greatest weights. Finally, the vegetable variety index is calculated by multiplying the Berry Index by the Health Value, which affirms that higher scores are attained by (1) the consumption of a greater number of vegetable subgroups, and (2) a greater consumption of subgroups that have greater recommended consumption amounts. Index scores range from 0.0 to 0.64.	Least vegetable variety, intermediate vegetable variety, greatest vegetable variety	Dark green vegetables, red and orange vegetables, legumes, starchy vegetables, and other vegetables	Least variety: 0.00, Intermediate variety: 0.25 (0.25-0.25), Greatest variety: 0.48 (0.48-0.48)
Kelger et al., 2021	*Dietary intake measurement tool*: survey, noted as a validated measure.<br><br>*Variety score*: An inventory of the number of fruits (out of 17 possible options) and vegetables (out of 22 possible options) consumed was calculated.	Odds ratio	Fruit (17 options, top 5 include, Apples, bananas, grapes, oranges, strawberries), and vegetables (22 options, top 5 include: onions, carrots, tomatoes, lettuce, broccoli).	Not applicable

Kobayashi et al. 2019	*Dietary intake measurement tool:* 138-item semi-quantitative FFQ (over the preceding year)<br><br>*Variety score:* If the consumption frequency of the same food or beverage was more than once per day, it was counted as once per day. Diversity was calculated as the number of items from the list consumed per day, and diversity within vegetables (24 items), and fruit (16 items).	Quintiles	Vegetables (24 items) and fruit (16 items), otherwise not specified.	Men/Vegetable (median freq):<br>Q1 (low): 1.7<br>Q2: 3.0<br>Q3: 4.2<br>Q4: 5.7<br>Q5 (high): 8.4<br><br>Men/Fruit (median freq):<br>Q1: 0.6<br>Q2: 1.3<br>Q3: 2.2<br>Q4: 3.4<br>Q5: 5.7<br><br>Women/Vegetable:<br>Q1: 2.3<br>Q2: 3.8<br>Q3: 5.2<br>Q4: 6.8<br>Q5: 9.4<br><br>Women/Fruit:<br>Q1: 2.0<br>Q2: 2.1<br>Q3: 3.3<br>Q4: 4.7<br>Q5: 7.1
Lamb et al., 2017	*Dietary intake measurement tool:* 130-item validated, semi-quantitative FFQ<br><br>*Variety score:* Variety of fruit and vegetable intake was derived by summing the total number of unique fruit and vegetable items consumed at least once per week. Possible variety ranged from 0 to 37 items. Potatoes were not included in the analyses, with the reasoning that they differ from vegetables in terms of energy and carbohydrate content and are commonly substituted for cereals rather than vegetables. Fruit juice, was also not included in the analyses, as it is not considered to be equivalent to whole fruit regarding fibre content and satiety value.	Continuous (regression coefficients)	Potatoes and fruit juices were not included, otherwise not specified.	Not applicable.

López-González et al., 2022	*Dietary intake measurement tool:* 143-item validated, semi-quantitative FFQ.<br><br>*Variety score:* The FFQ includes 13 and 17 items about fruit and vegetable intake, respectively. In each item, a typical portion size was defined as well as nine potential categories of fruit and vegetable frequency intake that varied from never or almost never to more than six times per day. The present study has been focused on solid and raw fruits and vegetables which are frequently consumed in Spain, so fruit and vegetable juices were excluded. Besides, as dried fruits refer to more than one type of fruit, they were also excluded because it was impossible to find out what type of raw fruit would be equivalent. Finally, potatoes and mushrooms were not considered vegetables because its nutritional composition differs from that of vegetables. Therefore, ten items of fruits such as oranges, bananas, apples, strawberries, cherries, melon, watermelon, kiwis, grapes, and peaches and eleven of vegetables including chards, cabbage, lettuce, tomatoes, carrots, green beans, courgette, peppers, asparagus, onions and garlics were finally incorporated. Variety of fruit and vegetable intake was measured as the sum of the total number of unique items consumed, regardless of quantity, which corresponds to the at least 1–3 per month response category in the FFQ. After that, continuous scores for variety in items consumed per month of fruits (0–10), vegetables (0–11) and both (0–21) were created at baseline, six months and 1-year of follow-up. This scoring method is similar to those used for reducing the risk of several chronic diseases in other cohort.	Beta-coefficient (continuous)	Solid and raw fruits and vegetables which are frequently consumed in Spain, so fruit and vegetable juices were excluded. Besides, as dried fruits refer to more than one type of fruit, they were also excluded. potatoes and mushrooms were not considered vegetables because its nutritional composition differs from that of vegetables. Therefore, ten items of fruits such as oranges, bananas, apples, strawberries, cherries, melon, watermelon, kiwis, grapes and peaches and eleven of vegetables including chards, cabbage, lettuce, tomatoes, carrots, green beans, courgette, peppers, asparagus, onions and garlics were finally included.	Variety (items/month)<br><br>Fruits and vegetables<br>Baseline: 15.44±3.42<br>6months: 15.47±3.32<br>1-year: 15.84±3.20<br><br>Fruits<br>Baseline: 7.71±2.26<br>6-months: 7.43±2.32<br>1-year: 7.77±2.20<br><br>Vegetables<br>Baseline: 7.73±2.04<br>6-months: 8.04±1.95<br>1-year: 8.07±1.93

Oude Griep et al., 2012	*Dietary intake measurement tool:* 178-item validated, semi-quantitative FFQ (over the preceding year)<br><br>*Variety score:* The fruit and vegetables assessed were those commonly consumed in the Netherlands. Fruit and vegetable consumption during winter and summer were assessed separately to consider seasonal variation. Fruit and vegetable juices and sauces were excluded, and potatoes and legumes were not considered as vegetables, because their nutritional value differs significantly from that of vegetables. The FFQ comprised 9 fruit items, 7 raw vegetables and 13 cooked vegetables. Each different fruit or vegetable that was consumed at least once per 2 weeks over the previous year contributed 1 point to the variety score. Several vegetable items that were essentially the same food but appeared in different forms, e.g., raw and cooked carrots, contributed only 1 point if their combined intake was at least once per 2 weeks. Several items were combined in single questions and could therefore not be distinguished from one another, i.e., apples and pears, cabbages, and leeks and onions. Variety scores ranged from 0 to 22 for fruit and vegetables together, from 0 to 9 for fruit, and from 0 to 13 for vegetables.	Tertiles	Fruit (9 items), raw vegetables (7 items), cooked vegetables (13 items). Several vegetable items that were essentially the same food but appeared in different forms, e.g., raw and cooked carrots, contributed only 1 point if their combined intake was at least once per 2 weeks. Several items were combined in single questions and could therefore not be distinguished from one another, i.e., apples and pears, cabbages, and leeks and onions.	T1 (mean±SD): 5.7±2.0<br>T2: 10.5±1.1<br>T3: 15.3±1.9
Yeung et al., 2021	*Dietary intake measurement tool:* 280-item validated, semi-quantitative FFQ<br><br>*Variety score*: The FFQ includes questions for the intake of specific fruits and vegetables. Potato, sweet potato, sweet corn, Chinese water chestnut, lotus root, pumpkin, taro, and preserved vegetables were not included in the calculation of total quantity and variety of vegetable intake, as their starch and/or sodium content is very different from other vegetables. Fruit cocktail, fruit juice with added sugar and canned fruits were not included in the calculation of total quantity and variety of fruit intake, as an abundant amount of sugar is often added. One point was given for each specific type of fruits and vegetables that was consumed at least a few times per year, regardless of the quantity. The possible ranges for the summed variety scores were from 0 to 25 for fruit intake, from 0 to 46 for vegetable intake and from 0 to 71 for combined fruit and vegetable intake.	Tertiles	Not included: potato, sweet potato, sweet corn, Chinese water chestnut, lotus root, pumpkin, taro, and preserved vegetables. Fruit cocktail, fruit juice with added sugar and canned fruits were not included in the calculation of total quantity and variety of fruit.	Fruit variety<br>T1: ≤6<br>T2: 7-11<br>T3: ≥12<br><br>Vegetable variety<br>T1: ≤20<br>T2: 21-27<br>T3: ≥28<br><br>Combined FV Variety<br>T1: ≤28<br>T2: 29-37<br>T3: ≥38

Abbreviations: DGA, Dietary Guidelines for Americans; FFQ, food frequency questionnaire; freq, frequency; FV, fruits and vegetables; Q, quantile; SD, standard deviation; T, tertile; USDA, United States Department of Agriculture.

Supplemental Table S8. Confounding variables of included cross-sectional studies.

Cohort	TLGS	FICSIT	BPRHS	NHANES
**Study**	Azadbakht et al., 2006	Bernstein et al., 2002	Bhupathiraju et al., 2011	Conrad et al., Nutr J 2018
Number of variables in fully adjusted [illegible]	14	4	16	11
**Pre-specified primary confounding variable**				
Energy intake	√	√	√	√
**Pre-specified secondary confounding variables**				
Age	√	√	√	√
Sex	√	√	√	√
Amount of VF Intake			√	√
Physical activity	√			
Smoking	√		√	√
Baseline BMI / Body Weight / WC	√	√	√	√
**Other confounding variables**				
Education				√
Household income			√	
Income-to-poverty ratio				√
Race/ethnicity				√
Waist-to-hip ratio	√			
Stress			√	
White blood cell count			√	
**Dietary Intake**				
Alcohol			√	
Sweets/desserts				√
Mediterranean diet adherence	√			
Trans fat			√	
Saturated fat			√	
Unsaturated: saturated fatty acids				√
Fat (%)	√			
Carbohydrate (%)	√			
Protein (%)	√			
Other food group/item	√			
**Disease History**				
Diabetes prevalence			√	
**Medications**				
Hormone replacement therapy use	√			
Vitamin supplement use			√	
Cardiovascular medication			√	
Diabetes medication			√	
Blood pressure-lowering	√			

Abbreviations: BPRHS = Boston Puerto Rican Health Study, BMI = body mass index, FICSIT = Frailty and Injuries: Cooperative Studies of Intervention Techniques, NHANES = National Health and Nutrition Examination Survey, TLGS = Tehran Lipid and Glucose Study, VF = vegetable/fruit, WC = waist circumference.

Supplemental Table S9. Confounding variables of included prospective cohort studies.

Cohort	NHS + HPFS	NHANES	JPHC	ADDITION	PREDIMED-Plus	MORGEN	Mr. OS & Ms. OS
**Study**	Bhupathiraju et al., 2013	Conrad et al.,	Kobayashi et al, 2019	Lamb et al., 2017	López-González et	Oude Griep et al., 2012	Yeung et al. 2021
Number of variables in fully adjusted model	16	11	12	13	16	13	17
**Pre-specified primary confounding variable**							
Energy intake	√	√	√	√	√	√	√
**Pre-specified secondary confounding variables**							
Age	√	√	√	√	√	√	√
Sex		√		√	√	√	√
Amount of VF Intake				√		√	√
Physical activity	√		√	√	√		√
Smoking	√	√	√	√	√	√	√
Baseline BMI/Body Weight/WC	√	√	√		√	√	√
**Other confounding variables**							
Education		√			√	√	√
Area of residence/ center			√		√		
Employment status					√		
Income-to-poverty ratio		√					
Occupational / socio-economic status				√			√
Occupation type			√				
Race/ethnicity		√		√			
Calendar year	√						
Solicitude			√				
Intervention group				√	√		
Lives alone					√		√
Marital status					√		√
Menopausal status	√						
CSID category							√
PASE score							√
DQI-I Score							√
**Dietary Intake**							
Alcohol	√		√	√	√	√	√
Whole grains						√	
Cereal fiber	√						
Sweets/desserts		√					
Processed meats						√	
Unprocessed red meats	√						
Seafood	√					√	
Trans fat	√						
Unsaturated: saturated fat		√					
Coffee			√				
Green tea			√				
Other food group/item			√				

Study	Bhupathiraju et al., 2013	Conrad et al.,	Kobayashi et al, 2019	Lamb et al., 2017	López-González et	Oude Griep et al., 2012	Yeung et al. 2021
**Disease History**							
Diabetes prevalence					√		
Hypertension prevalence					√		
Hypercholesterolemia prevalence					√		
Family history of acute myocardial infarction	√					√	
Depressive symptoms							√
Number of chronic diseases							√
**Medications**							
Hormone replacement therapy use	√					√	
Vitamin supplement use	√					√	
Cardiometabolic medication		√					
Aspirin use	√						
Blood pressure-lowering				√			
Glucose-lowering medication				√			
Lipid-lowering medication				√			

Abbreviations: ADDITION = Anglo–Danish–Dutch Study of Intensive Treatment In People with Screen-Detected Diabetes in Primary Care-Cambridge study, BMI = body mass index, HPFS = Health Professionals Follow-up Study, JPHC = Japan public health center study, MORGEN = The Monitoring Project on Risk Factors and Chronic Diseases in the Netherlands Study, NHANES = National Health and Nutrition Examination Survey, NHS = Nurses Health Study, PREDIMED-Plus = PREvención con DIeta MEDiterránea (Prevention with Mediterranean Diet)-Plus, VF = vegetable/fruit.

Supplemental Table S10. Risk of bias scores of included observational cohort studies.

Supplemental Table S10a. Study Quality Assessment Tool for Observational Cohort and Cross-sectional Studies from the National Heart, Lung, and Blood Institute scores of included cross-sectional studies.

**Reference (Last name et al., Year)**	1.	2.	3.	4.	5.	6.	7.	8.	9.	10.	11.	12.	13.	14.	Overall Quality Rating	Total
Azadbakht et al., 2006	1	1	0	1	0	NA	NA	1	1	NA	1	0	NA	1	Fair	7
Bernstein et al., 2002	1	1	0	1	0	NA	NA	0	1	NA	1	0	NA	1	Fair	6
Bhupathiraju et al., 2011	1	1	1	1	0	NA	NA	1	1	NA	1	0	NA	0	Fair	7
Conrad et al., 2018	1	1	NA	1	0	NA	NA	1	1	NA	0	0	NA	1	Fair	6
Kegler et al., 2021	1	1	1	1	0	NA	NA	1	1	NA	0	0	NA	1	Fair	7

1. Was the research question or objection in this paper clearly stated?
2. Was the study population clearly specified and defined?
3. Was the participation rate of eligible persons at least 50%?
4. Were all the participants selected or recruited from the same or similar populations (including the same time period)? Were inclusion and exclusion criteria for being in the study prespecified and applied uniformly to all participants?
5. Was a sample size justification, power description, or variance and effect estimates provided?
6. For the analyses in the paper, were the exposure(s) of interest measured prior to the outcome(s) being measured?
7. Was the timeframe sufficient so that one could reasonably expect to see an association between exposure and outcome if it existed?
8. For exposures that can vary in amount or level, did the study examine different levels of the exposure as related to the outcome (e.g., categories or exposure, or exposure measured as continuous variable)?
9. Were the exposure measures (independent variables) clearly defined, valid, reliable, and implemented consistently across all study participants?
10. Was the exposure(s) assessed more than once over time?
11. Were the outcome measures (dependent variables) clearly defined, valid, reliable, and implemented consistently across all study participants?
12. Were the outcome assessors blinded to the exposure status of participants?
13. Was loss to follow-up after baseline 20% or less?
14. Were key potential confounding variables measured and adjusted statistically for their impact on the relationship between exposure(s) and outcome(s)?
15. Overall Quality Rating (Good, Fair, or Poor)

Abbreviations: NA, not available or applicable.

Supplemental Table S10b. Newcastle-Ottawa Scale (NOS) scores of included prospective cohort studies.

Reference (Last name et al., Year)	Selection (max 4)[a]				Outcome (max 3)[b]			Comparability (max 2)[c]		Total[d]
	Representativeness of the exposed cohort	Selection of the non-exposed cohort	Ascertainment of exposure	Demonstration that outcome of interest was not present at start of study	Assessment of outcome	Was follow-up long enough for outcomes to occur	Adequacy of follow-up of cohort	Study controls for energy	Study controls for pre-specified secondary covariates	
Bhupathiraju et al., 2013	0	1	1	1	1	1	1	1	1	8
Conrad et al., 2018	1	1	1	0	1	1	1	1	1	8
Kobayashi et al, 2019	1	1	1	1	1	1	1	1	1	9
Lamb et al., 2017	0	1	1	0	1	1	0	1	1	6
López-González et al., 2022	1	1	1	0	1	1	1	1	1	8
Oude Griep et al., 2012	1	1	1	1	1	1	1	1	1	9
Yeung et al. 2021	1	1	1	1	1	1	0	1	1	8

[a]Maximum 4 points awarded for cohort representativeness, selection of non-exposed cohort, exposure assessment, and demonstration that the outcome is not present at baseline

[b]Maximum 3 points awarded for follow-up length, adequacy of follow-up, and outcome assessment

[c]Maximum 2 points awarded for controlling for the pre-specified primary confounding variable (energy intake) and 4 of the 6 secondary (age, sex, amount of vegetable and/or fruit intake, physical activity, smoking status, baseline BMI or body weight) confounding variables

[d]A maximum of 9 points could be awarded. Cohorts with NOS ≥6 are considered high quality.

Abbreviations: BMI = body mass index; NOS = Newcastle-Ottawa Scale.

Supplemental Table S11. GRADE assessment.

Outcome and trial (N)	Design	Risk of bias (ROB)	Inconsistency	Indirectness	Imprecision	Publication bias	Dose response	Attenuation	Magnitude	Effect (RR | OR | MD [95% CI], P_{MD})	Certainty of Evidence[a]	Interpretation of magnitude of effect[b]
		**GRADE assessment**										
		**Downgrades**					**Upgrade**					
**Mortality**												
All-cause (7)	Prospective Cohorts	Not serious	Serious[1]	Not serious	Serious[2]	None[3]	None[4]	None	None	↓ 0.89 [0.82 to 0.97], P=0.007	⊕◯◯◯ Very low	Trivial
CVD (7)	Prospective Cohorts	Not serious	Not serious	Not serious	Serious[5]	None[3]	None[4]	None	None	↔ 0.90 [0.80 to 1.01], P=0.150	⊕◯◯◯ Very low	No effect
CHD (2)	Prospective Cohorts	Not serious	Serious[6]	Not serious	Serious[7]	None[3]	None[4]	None	None	↔ 0.97 [0.86 to 1.09], P=0.620	⊕◯◯◯ Very low	No effect
**Incidence**												
CHD (4)	Prospective Cohorts	Not serious	Not serious	Not serious	Not serious	None[3]	None[4]	None	None	↔ 1.04 [0.96 to 1.12], P=0.34	⊕⊕◯◯ Low	No effect
Stroke (2)	Prospective Cohorts	Not serious	Not serious	Not serious	Serious[8]	None[3]	None[4]	None	None	↔ 0.84 [0.63 to 1.13], P=0.25	⊕◯◯◯ Very low	No effect
**Prevalence**												
CVD (2)	Cross-sectional	Not serious	Not serious	Not serious	Serious[9]	None[3]	None[4]	None	None	↔ 0.86 [0.67 to 1.09], P=0.21	⊕◯◯◯ Very low	No effect
CHD (2)	Cross-sectional	Not serious	Not serious	Not serious	Serious[10]	None[3]	None[4]	None	None	↔ 0.80 [0.57 to 1.11], P=0.19	⊕◯◯◯ Very low	Trivial
Stroke (2)	Cross-sectional	Not serious	Not serious	Not serious	Serious[11]	None[3]	None[4]	None	None	↔ 1.01 [0.75 to 1.35], P=0.97	⊕◯◯◯ Very low	No effect
**Risk Factors**												
Blood Lipids												
HCL (1)	Cross-sectional	Not serious	Not serious[12]	Not serious	Not serious	None[3]	None[4]	None	None	↓ 0.68 [0.58 to 0.81], P<0.05	⊕⊕◯◯ Low	Trivial
High LDL-C (1)	Cross-sectional	Not serious	Not serious[12]	Not serious	Serious[13]	None[3]	None[4]	None	None	↔ 0.80 [0.57 to 1.13], P=0.203	⊕◯◯◯ Very low	No effect
LDL-C (1)	Prospective Cohorts	Not serious	Not serious[12]	Not serious	Serious[13]	None[3]	None[4]	None	None	↔ 0.37 [-15.36 to 16.09], P=0.96	⊕◯◯◯ Very low	No effect

Low HDL-C (1)	Cross-sectional	Not serious	Not serious[12]	Not serious	Serious[13]	None[3]	None[4]	None	None	↔	0.94 [0.70 to 1.27], P=0.700	⊕◯◯◯ Very low	No effect
HDL-C (3)	Prospective Cohorts	Serious[1]	Not serious	Not serious	Serious[13]	None[3]	None[4]	None	None	↔	-0.02 [-0.30 to 0.26], P=0.89	⊕◯◯◯ Very low	No effect
HTG (1)	Cross-sectional	Not serious	Not serious[12]	Not serious	Serious[14]	None[3]	None[4]	None	None	↔	0.83 [0.59 to 1.16], P=0.265	⊕◯◯◯ Very low	No effect
TG (3)	Prospective Cohorts	Serious[1]	Not serious	Not serious	Serious[13]	None[3]	None[4]	None	None	↔	0.06 [-1.11 to 1.22], P=0.92	⊕◯◯◯ Very low	No effect
Blood Pressure													
HTN (1)	Cross-sectional	Not serious	Not serious[12]	Not serious	Serious[15]	None[3]	None[4]	None	None	↔	0.76 [0.55 to 1.04], P=0.083	⊕◯◯◯ Very low	No effect
SBP (3)	Prospective Cohorts	Serious[1]	Not serious	Not serious	Serious[16]	None[3]	None[4]	None	None	↔	0.11 [-11.41 to 11.64], P=0.98	⊕◯◯◯ Very low	No effect
DBP (1)	Prospective Cohorts	Not serious	Not serious[12]	Not serious	Serious[16]	None[3]	None[4]	None	None	↔	0.14 [-7.75 to 8.03], P=0.97	⊕◯◯◯ Very low	No effect
Glycemic Control													
Diabetes (3)	Cross-sectional	Not serious	Not serious	Not serious	Serious[17]	None[3]	None[4]	None	None	↔	1.02 [0.84 to 1.24], P=0.804	⊕◯◯◯ Very low	No effect
HbA1c (2)	Prospective Cohorts	Serious[5]	Not serious	Not serious	Serious[18]	None[3]	None[4]	None	None	↔	0.02 [-1.18 to 1.22], P=0.97	⊕◯◯◯ Very low	No effect
Fasting Glucose (1)	Prospective Cohorts	Not serious	Not serious[12]	Not serious	Serious[19]	None[3]	None[4]	None	None	↔	-0.68 [-20.20 to 18.85], P=0.95	⊕◯◯◯ Very low	No effect
Adiposity													
Obesity (1)	Cross-sectional	Not serious	Not serious[12]	Not serious	Not serious	None[3]	None[4]	None	None	↓	0.72 [0.59 to 0.88], P<0.05	⊕⊕◯◯ Low	Trivial
Body Weight (1)	Prospective Cohorts	Not serious	Not serious[12]	Not serious	Serious[20]	None[3]	None[4]	None	None	↔	-0.14 [-4.59 to 4.31], P=0.95	⊕◯◯◯ Very low	No effect
WC (3)	Prospective Cohorts	Serious[1]	Not serious	Not serious	Serious[21]	None[3]	None[4]	None	None	↔	0.12 [-4.94 to 5.18], P=0.96	⊕◯◯◯ Very low	No effect
Inflammation													
CRP (1)	Cross-sectional	Not serious	Not serious[12]	Not serious	Not serious	None[3]	None[4]	None	None	↔	-0.01 [-0.13 to 0.11], P=0.89	⊕⊕◯◯ Low	No effect

Risk Scores													
CCMR (2)	Prospective Cohorts	Serious[5]	Not serious	Not serious	Not serious	None[3]	None[4]	None	None	↔	0.02 [-0.48 to 0.52], P=0.94	⊕⊕○○ Low	No effect
FRS (1)	Cross-sectional	Not serious	Not serious[12]	Not serious	Serious[22]	None[3]	None[4]	None	None	↔	-0.12 [-2.18 to 1.94], P=0.91	⊕○○○ Very low	No effect

[a] Since all included studies were observational cohorts, the certainty of the evidence was graded as low for all outcomes by default and then downgraded or upgraded based on pre-specified criteria. Criteria for downgrades included risk of bias (ROB) (downgraded if the majority of trials were considered to be at high ROB); inconsistency (downgraded if there was substantial unexplained heterogeneity [$I^2 \geq 50\%$, $P_Q < 0.10$]; indirectness (downgraded if there were factors absent or present relating to the participants, interventions, or outcomes that limited the generalizability of the results); imprecision (downgraded if the 95% confidence interval crossed the minimally important difference [MID] for harm or benefit set at RR=0.05 for the mortality, prevalence and incidence related outcomes, 0.1 mmol/L for lipids [12–14], 2 mm Hg for systolic and diastolic blood pressure [15], 0.3% for HbA1c [16], 0.5 mmol/L for fasting glucose [17], 0.5 kg for body weight, 2 cm for waist circumference, [18,19], 0.5 mg/L for CRP [20–22], 5% change for CCMR, 0.65 points for FRS [23]; and publication bias (downgraded if there were more than 10 comparisons and there is evidence of publication bias based on funnel plot asymmetry and/or significant Egger's or Begg's tests (P<0.10) with confirmation by adjustment by Duval and Tweedie trim-and-fill analysis). Criteria for upgrades included a significant dose-response gradient, attenuation, and large magnitude of effect.
[b] For the interpretation of the magnitude, MIDs were used (see a above) to assess the importance of magnitude of our point estimate using the effect size categories according to new GRADE guidance. MIDs were then used to assess the importance of the magnitude of our point estimates using the effect size categories according GRADE guidance [24–26] as follows: large effect (≥5x MID); moderate effect (≥2x MID); small important effect (≥1x MID); and trivial/unimportant effect (< 1 MID).

Abbreviations: BMI, body mass index; CCMR, clustered cardiometabolic risk scores; CI, confidence interval; DBP, diastolic blood pressure; DR, dose response; GRADE, Grading of Recommendations Assessment Development and Evaluation; HbA1c, hemoglobin A1c; HCL = hypercholesterolemia; HDL-C, high-density lipoprotein-cholesterol; HTG = hypertriglyceridemia; HTN = hypertension; LDL-C, low-density lipoprotein-cholesterol; MD, mean difference; MID, minimally important difference; OR, odds ratio; ROB, risk of bias; RR, risk ratio; SBP, systolic blood pressure; SMD, standardized mean difference; WC, waist circumference.

[1] Downgrade for serious inconsistency as there was evidence of substantial inter-study heterogeneity (I^2=68.5%, P=0.004).
[2] Downgrade for serious imprecision, as the lower bound of the 95% CI (HR, 0.82) includes the MID of 5% while the upper bound of the 95% CI (HR, 0.97) crosses the MID (RR=0.05).
[3] No downgrade for publication bias, as publication bias could not be assessed due to lack of power for assessing funnel plot asymmetry and small study effects (<10 trial comparisons included in the meta-analysis).
[4] No upgrade for dose-response, as dose-response could not be assessed due to lack of appropriate data for analysis.
[5] Downgrade for serious imprecision, as the lower bound of the 95% CI (HR, 0.83) includes the MID of 5% while the upper bound of the 95% CI (HR, 1.03) crosses the MID (RR=0.05).
[6] Downgrade for serious inconsistency as there was evidence of substantial inter-study heterogeneity (I^2=78.7%, P=0.03).

[7] Downgrade for serious imprecision, as the lower bound of the 95% CI (HR, 0.47) includes the MID of 5% while the upper bound of the 95% CI (HR, 0.98) crosses the MID (RR=0.05).
[8] Downgrade for serious imprecision, as the lower and upper bound of the 95% CIs (HR, 0.63 to 1.13) includes both clinically important benefit (HR<0.95) and harm (HR≥1.05).
[9] Downgrade for serious imprecision, as the lower bound of the 95% CI (RR, 0.67) includes the MID of 5% while the upper bound of the 95% CI (RR, 1.09) crosses the MID (RR=0.05).
[10] Downgrade for serious imprecision, as the lower bound of the 95% CI (RR, 0.57) includes the MID of 5% while the upper bound of the 95% CI (RR, 1.11) crosses the MID (RR=0.05).
[11] Downgrade for serious imprecision, as the lower bound of the 95% CI (RR, 0.75) includes the MID of 5% while the upper bound of the 95% CI (RR, 1.35) crosses the MID (RR=0.05).
[12] No downgrade for inconsistency as analyses for inconsistency could not be performed due to <2 observations available.
[13] Downgrade for serious imprecision as the 95% CI overlaps the MID of clinically important harm or benefit for lipids (0.1 mmol/L).
[14] Downgrade for serious imprecision as the lower bound of the 95% CI (RR, 0.59) includes the MID of 5% while the upper bound of the 95% CI (RR, 1.16) crosses the MID (RR=0.05).
[15] Downgrade for serious imprecision as the lower bound of the 95% CI (RR, 0.55) includes the MID of 5% while the upper bound of the 95% CI (RR, 1.04) crosses the MID (RR=0.05).
[16] Downgrade for serious imprecision as the 95% CI overlaps the MID of clinically important benefit for blood pressure (2 mm Hg).
[17] Downgrade for serious imprecision as the lower bound of the 95% CI (RR, 0.84) includes the MID of 5% while the upper bound of the 95% CI (RR, 1.24) crosses the MID (RR=0.05).
[18] Downgrade for serious imprecision as the 95% CI overlaps the MID of clinically important benefit for HbA1c (0.3%).
[19] Downgrade for serious imprecision as the 95% CI overlaps the MID of clinically important benefit for fasting glucose (0.5 mmol/L).
[20] Downgrade for serious imprecision as the 95% CI overlaps the MID of clinically important benefit for body weight (0.5 kg).
[21] Downgrade for serious imprecision as the 95% CI overlaps the MID of clinically important benefit for waist circumference (2 cm).
[22] Downgrade for serious imprecision as the 95% CI overlaps the MID of clinically important benefit for FRS (0.65 points).

Supplementary Figures

Supplemental Figure S1. Forest plot of prospective cohort studies of the association between variety of vegetable and/or fruit intake and all-cause mortality.

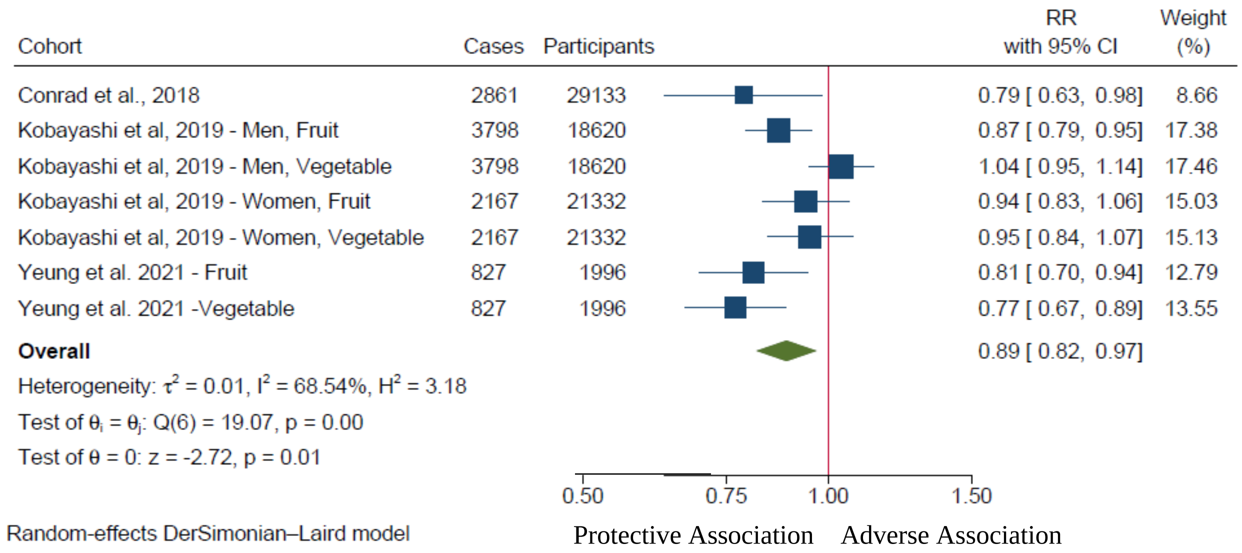

CI = confidence interval; RR = risk ratio.

Supplemental Figure S2. Forest plot of prospective cohort studies of the association between variety of vegetable and/or fruit intake and CVD mortality.

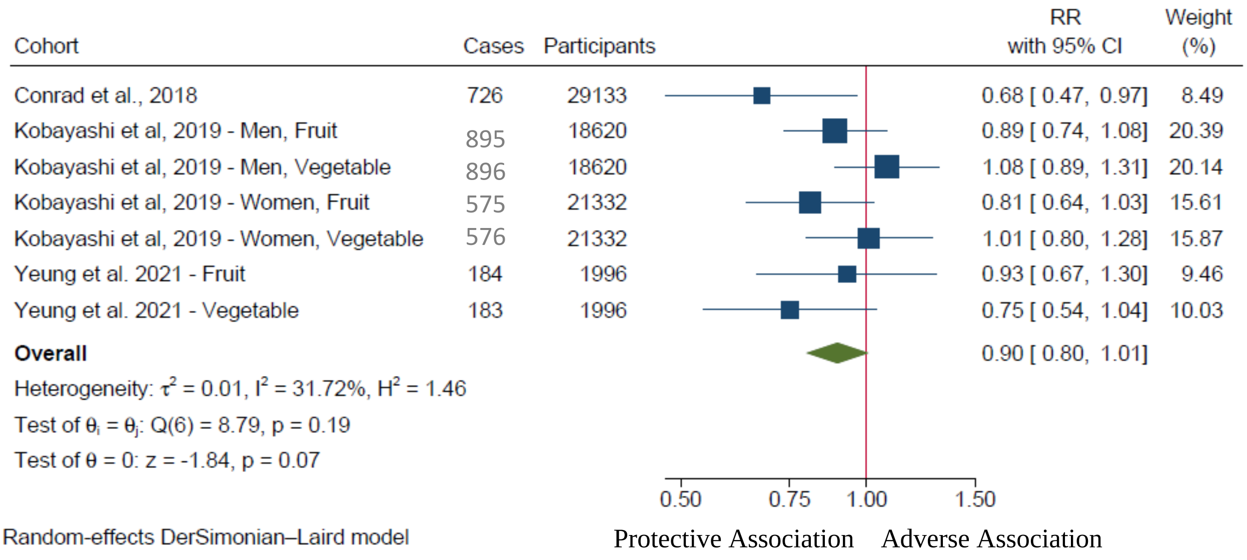

CI = confidence interval; CVD = cardiovascular disease; RR = risk ratio.

Supplemental Figure S3. Forest plot of prospective cohort studies of the association between variety of vegetable and/or fruit intake and CHD mortality.

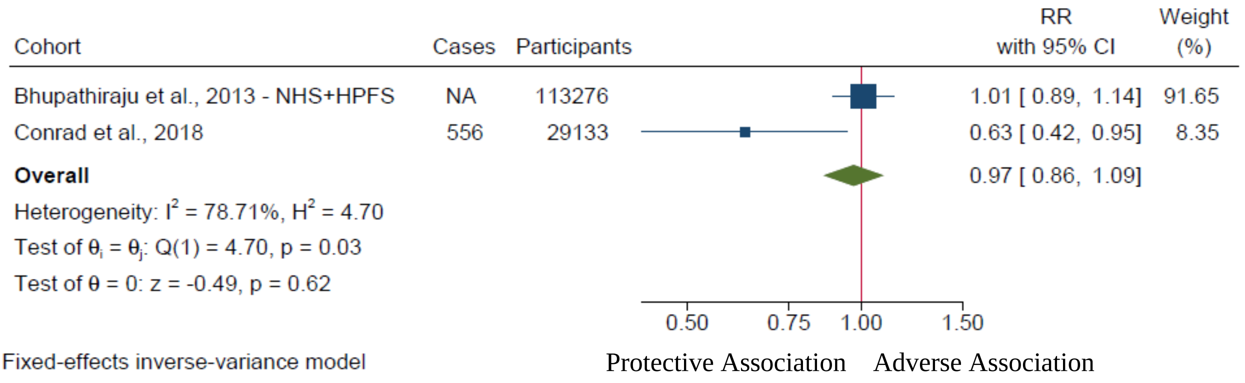

CHD =coronary heart disease; CI = confidence interval; RR = risk ratio.

Supplemental Figure S4. Forest plot of prospective cohort studies of the association between variety of vegetable and/or fruit intake and CHD incidence.

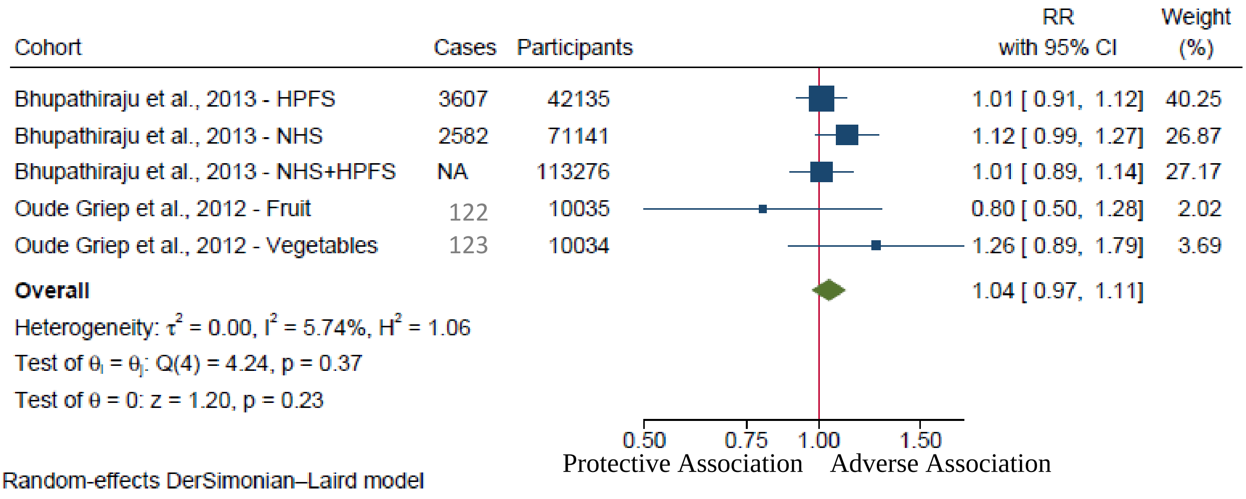

CHD = coronary heart disease; CI = confidence interval; HPFS = Health Professionals Follow-up Study; NHS = Nurses´ Health Study; RR = risk ratio.

Supplemental Figure S5. Forest plot of prospective cohort studies of the association between variety of vegetable and/or fruit intake and stroke incidence.

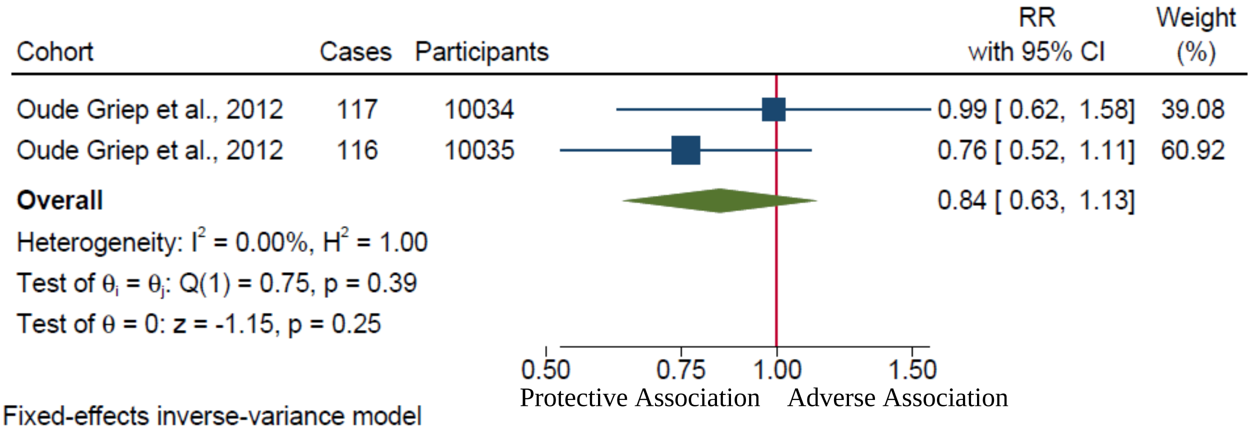

CI = confidence interval; RR = risk ratio.

Supplemental Figure S6. Forest plot of cross-sectional cohort studies of the association between variety of vegetable and/or fruit intake and CVD prevalence.

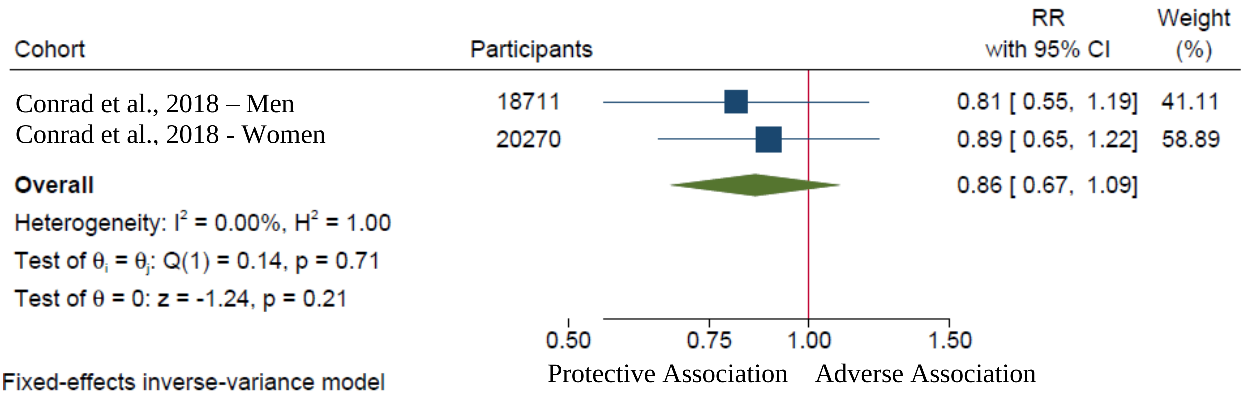

CI = confidence interval; CVD = cardiovascular disease; RR = risk ratio.

Supplemental Figure S7. Forest plot of cross-sectional cohort studies of the association between variety of vegetable and/or fruit intake and CHD prevalence.

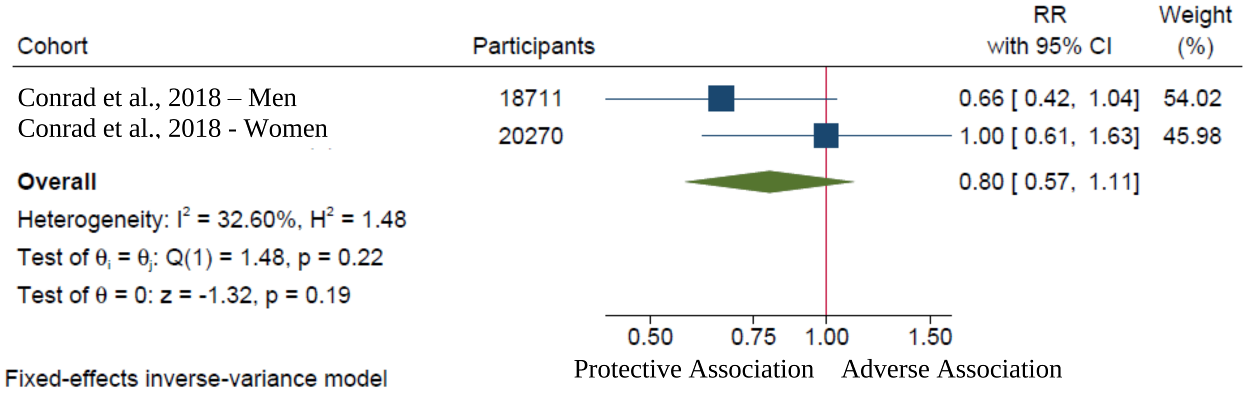

CHD = coronary heart disease; CI = confidence interval; RR = risk ratio.

Supplemental Figure S8. Forest plot of cross-sectional cohort studies of the association between variety of vegetable and/or fruit intake and stroke prevalence.

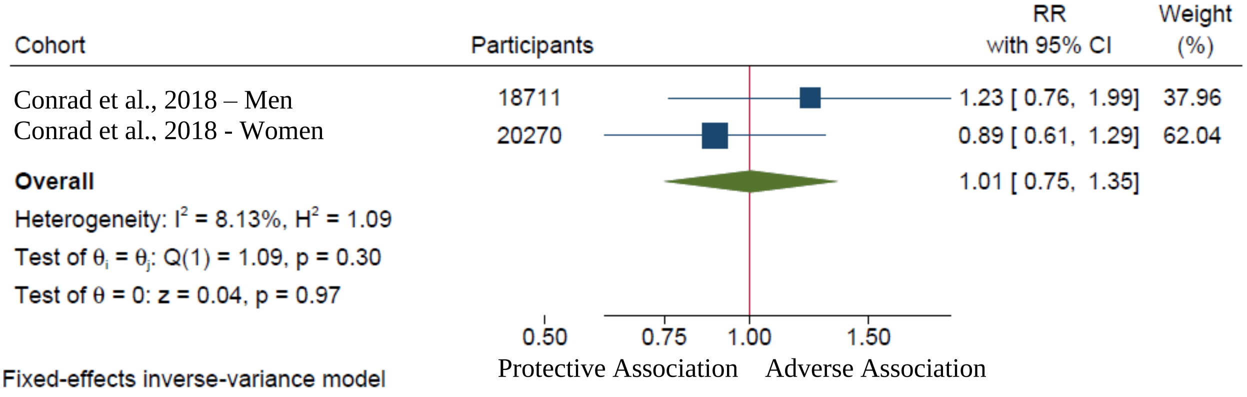

CHD = coronary heart disease; CI = confidence interval; RR = risk ratio.

Supplemental Figure S9. Forest plot of prospective cohort studies of the association between variety of vegetable and/or fruit intake and hypercholesterolemia.

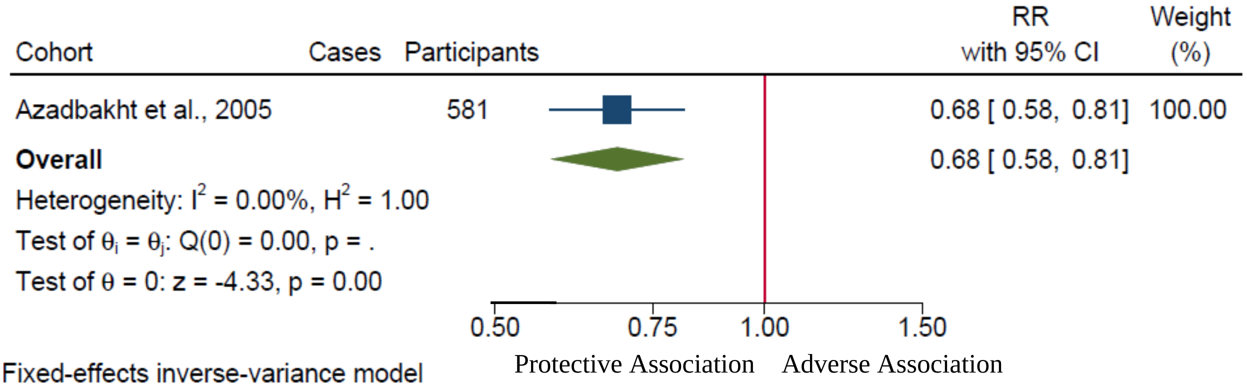

CI = confidence interval; RR = risk ratio.

Supplemental Figure S10. Forest plot of prospective cohort studies of the association between variety of vegetable and/or fruit intake and high LDL-C.

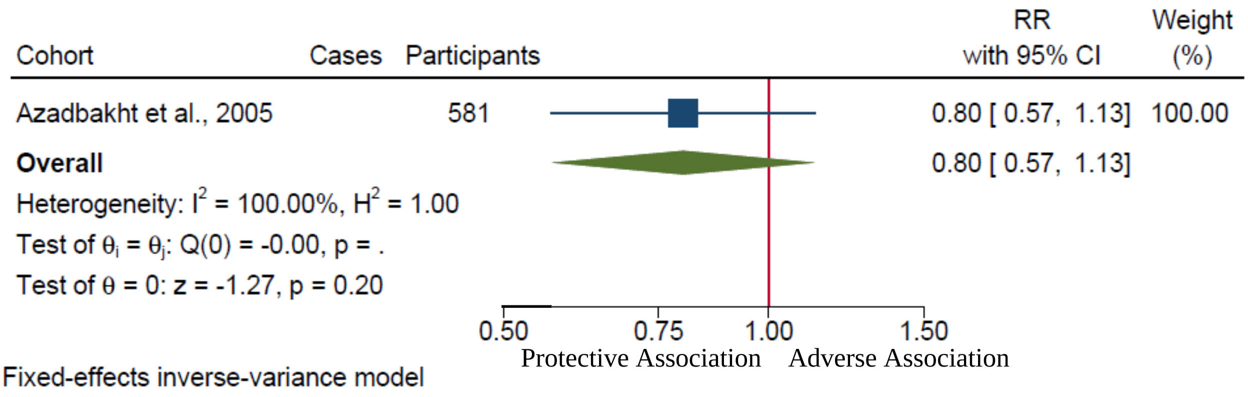

High LDL-C refers to an LDL-C level greater than or equal to 4.1 mmol/L.
CI = confidence interval; LDL-C = low-density lipoprotein-cholesterol; RR = risk ratio.

Supplemental Figure S11. Forest plot of prospective cohort studies of the association between variety of vegetable and/or fruit intake and LDL-C.

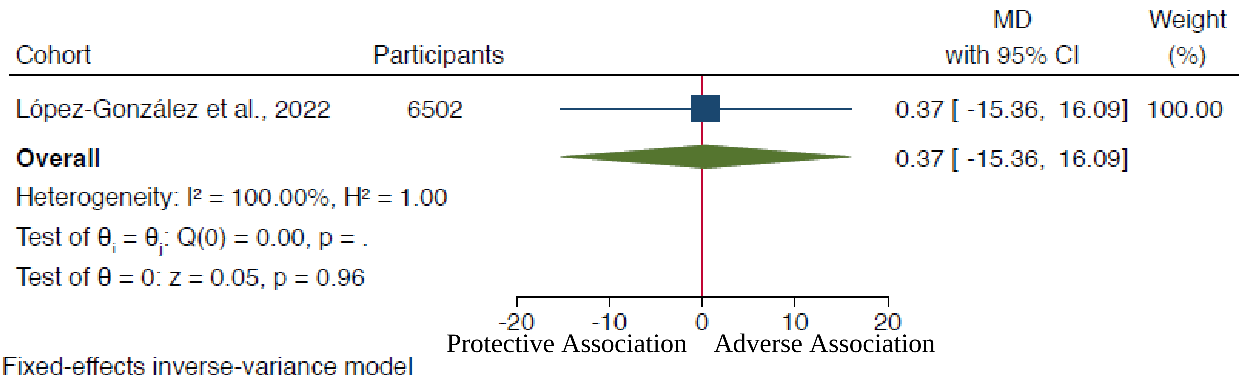

CI = confidence interval; LDL-C = low-density lipoprotein-cholesterol; MD = mean difference.

Supplemental Figure S12. Forest plot of prospective cohort studies of the association between variety of vegetable and/or fruit intake and low HDL-C.

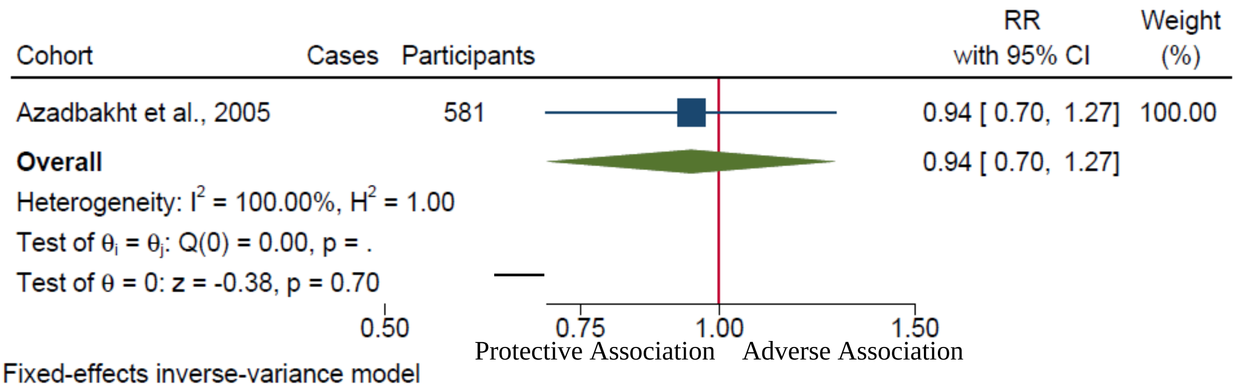

Low HDL-C refers to an HDL-C level less than 1.03 mmol/L.
CI = confidence interval; HDL-C = high-density lipoprotein-cholesterol; RR = risk ratio.

Supplemental Figure S13. Forest plot of prospective cohort studies of the association between variety of vegetable and/or fruit intake and HDL-C.

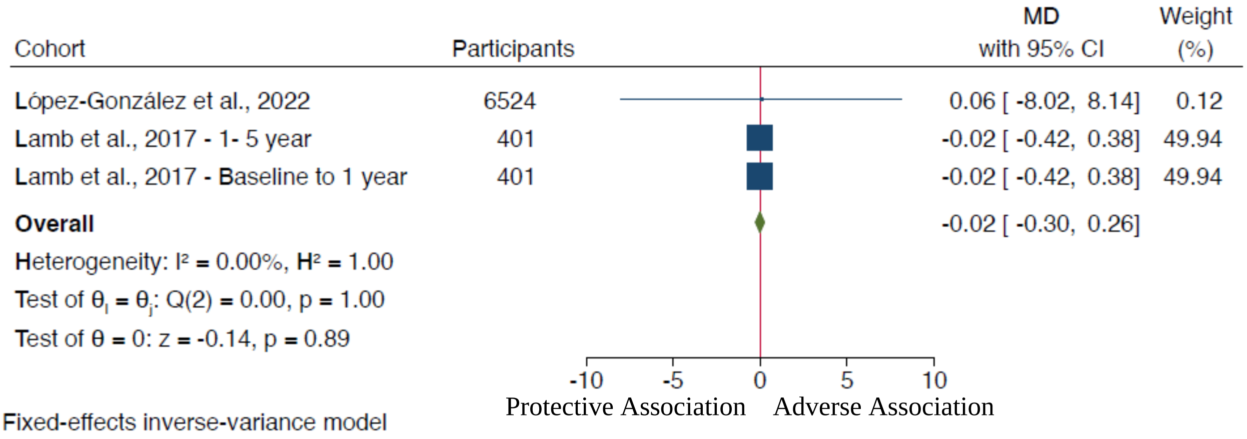

CI = confidence interval; HDL-C = high-density lipoprotein-cholesterol; MD = mean difference.

Supplemental Figure S14. Forest plot of prospective cohort studies of the association between variety of vegetable and/or fruit intake and hypertriglyceridemia.

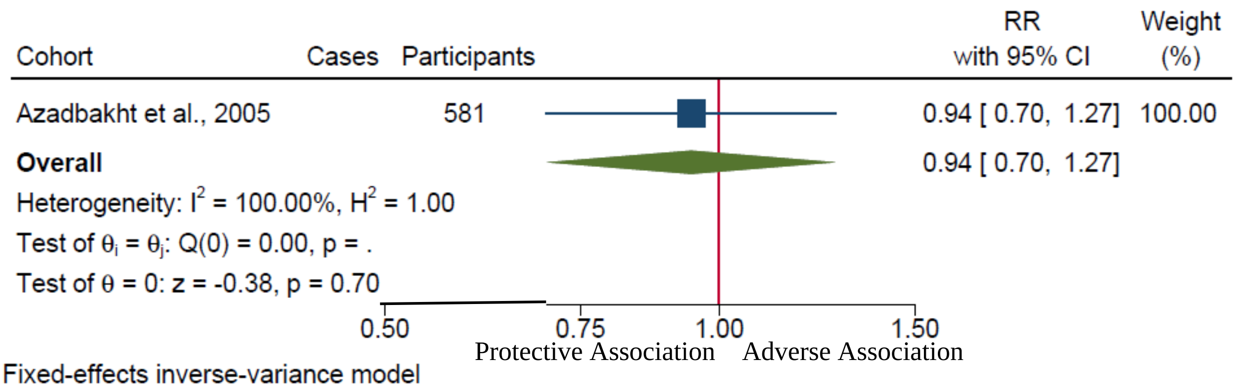

Hypertriglyceridemia refers to a triglyceride level greater than or equal to 2.3 mmol/L.
CI = confidence interval; RR = risk ratio.

Supplemental Figure S15. Forest plot of prospective cohort studies of the association between variety of vegetable and/or fruit intake and triglycerides.

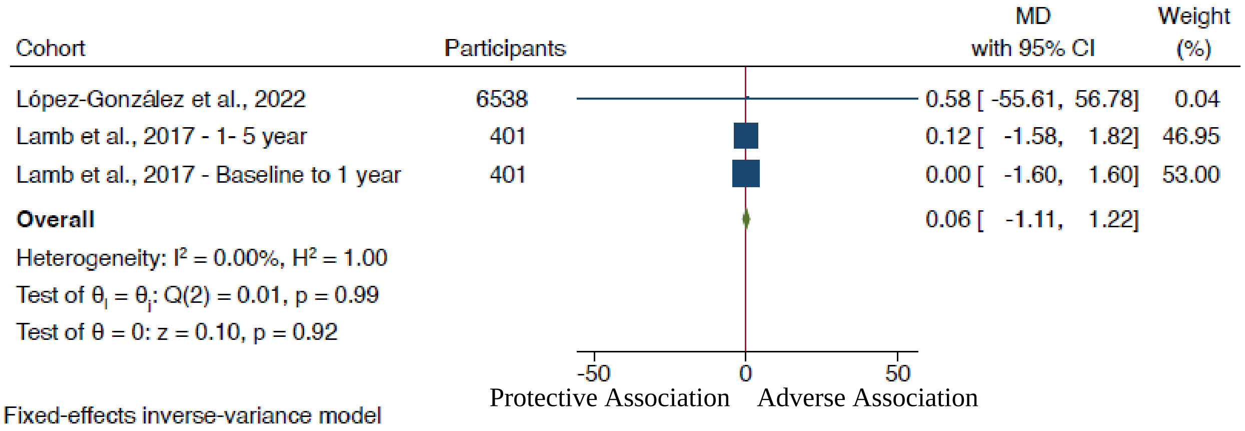

CI = confidence interval; MD = mean difference.

Supplemental Figure S16. Forest plot of cross-sectional cohort studies of the association between variety of vegetable and/or fruit intake and hypertension.

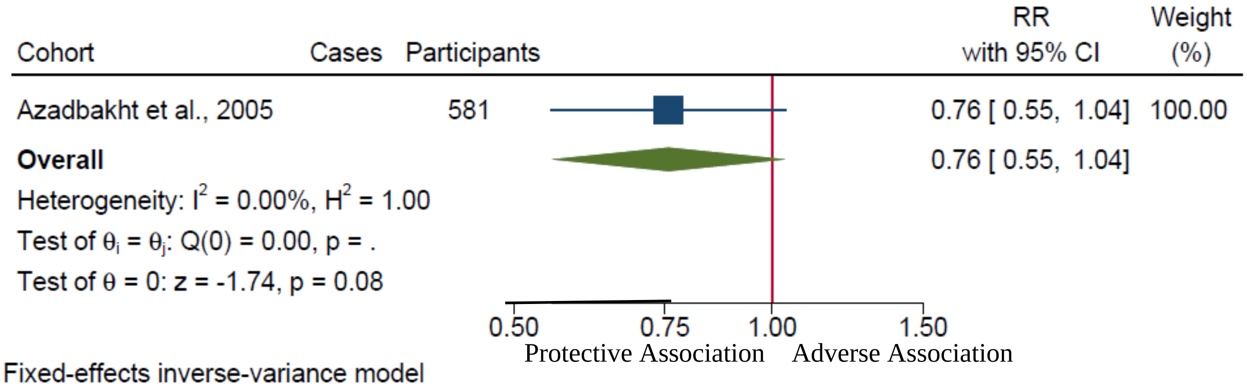

Hypertension refers to a systolic blood pressure ≥140 mm Hg or diastolic blood pressure ≥90 mm Hg.
CI = confidence interval; RR = risk ratio.

Supplemental Figure S17. Forest plot of prospective cohort studies of the association between variety of vegetable and/or fruit intake and systolic blood pressure.

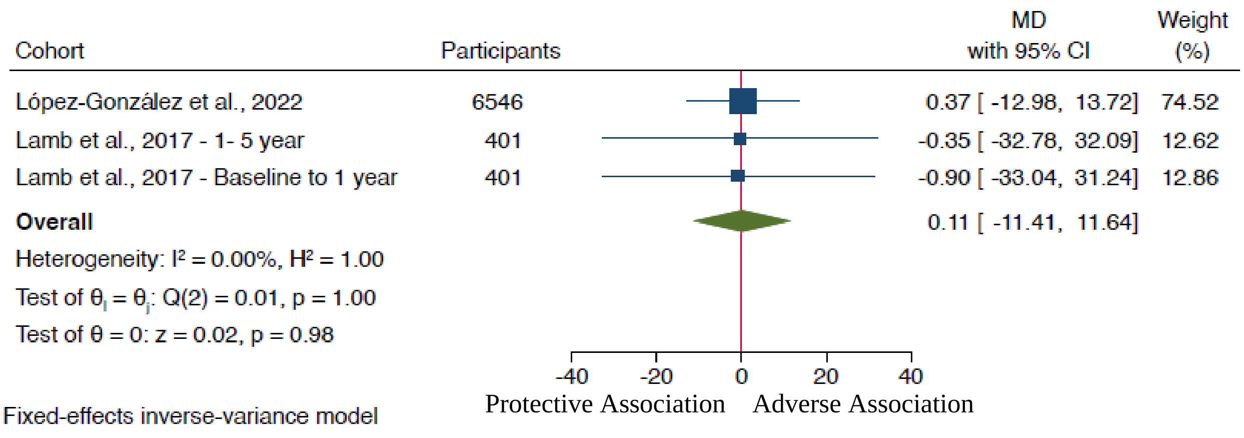

CI = confidence interval; MD = mean difference.

Supplemental Figure S18. Forest plot of prospective cohort studies of the association between variety of vegetable and/or fruit intake and diastolic blood pressure.

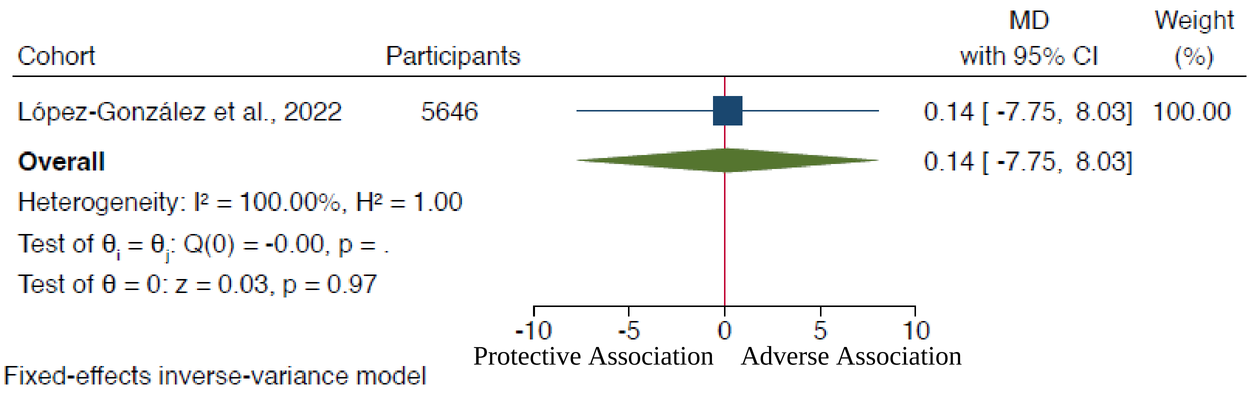

CI = confidence interval; MD = mean difference.

Supplemental Figure S19. Forest plot of cross-sectional studies of the association between variety of vegetable and/or fruit intake and obesity.

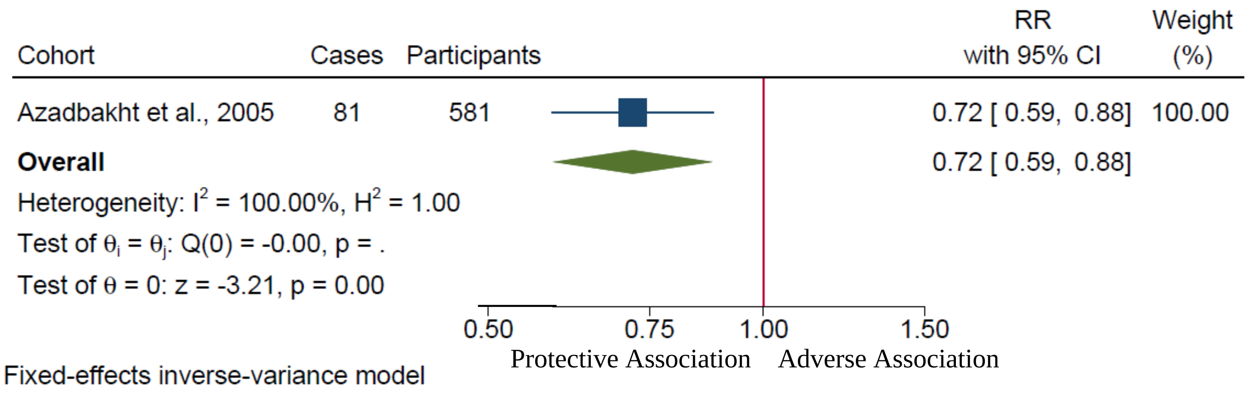

Obesity refers to a body mass index ≥30 kg/m^2.
CI = confidence interval; RR = risk ratio.

Supplemental Figure S20. Forest plot of prospective cohort studies of the association between variety of vegetable and/or fruit intake and body weight.

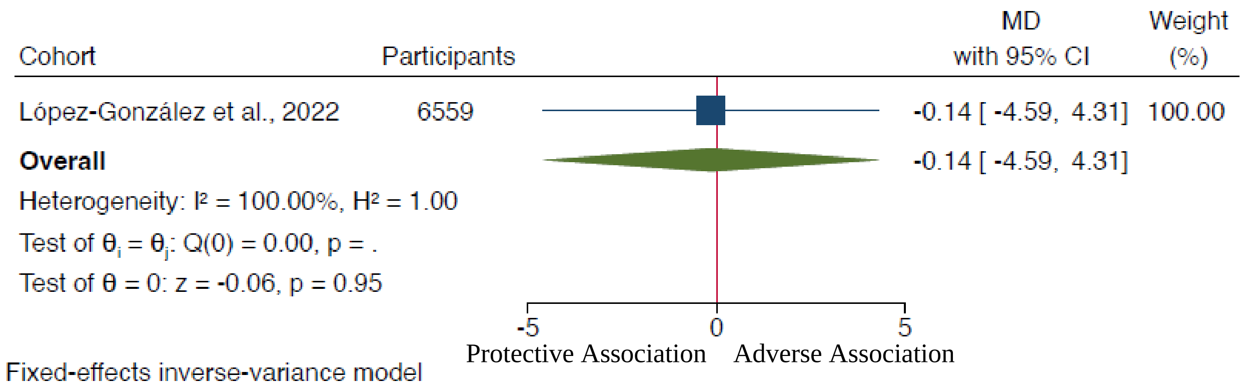

CI = confidence interval; MD = mean difference.

Supplemental Figure S21. Forest plot of prospective cohort studies of the association between variety of vegetable and/or fruit intake and waist circumference.

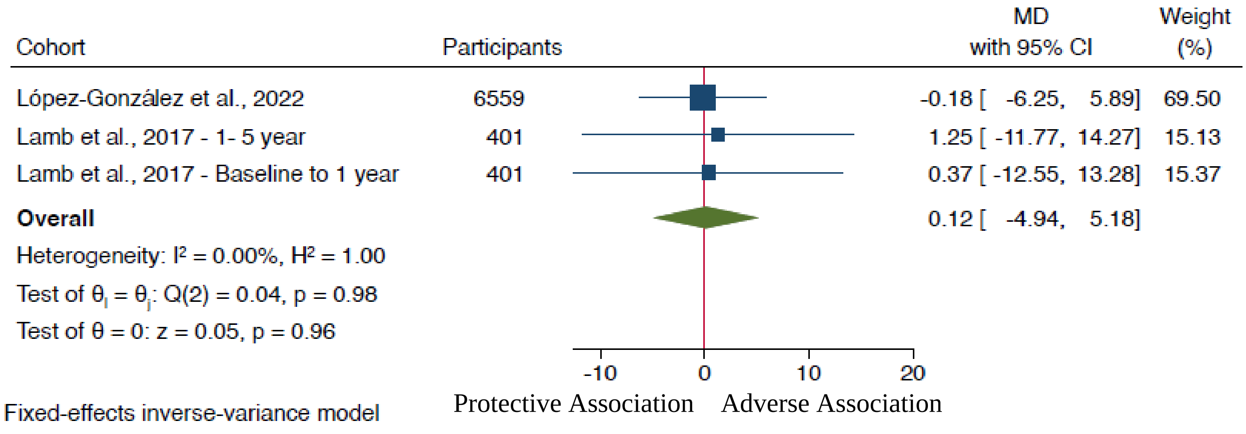

CI = confidence interval; MD = mean difference.

Supplemental Figure S22. Forest plot of prospective cohort studies of the association between variety of vegetable and/or fruit intake and C-reactive protein.

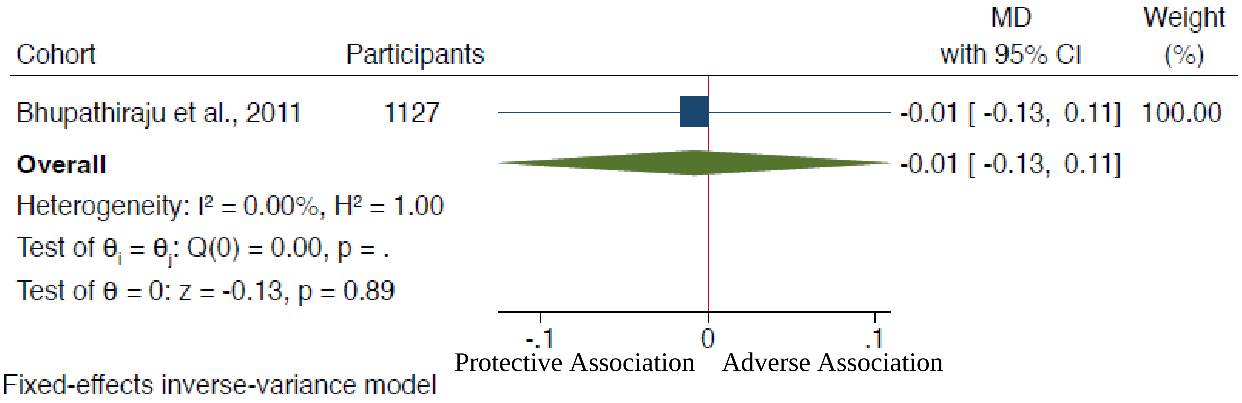

CI = confidence interval; MD = mean difference.

Supplemental Figure S23. Forest plot of cross-sectional studies of the association between variety of vegetable and/or fruit intake and diabetes.

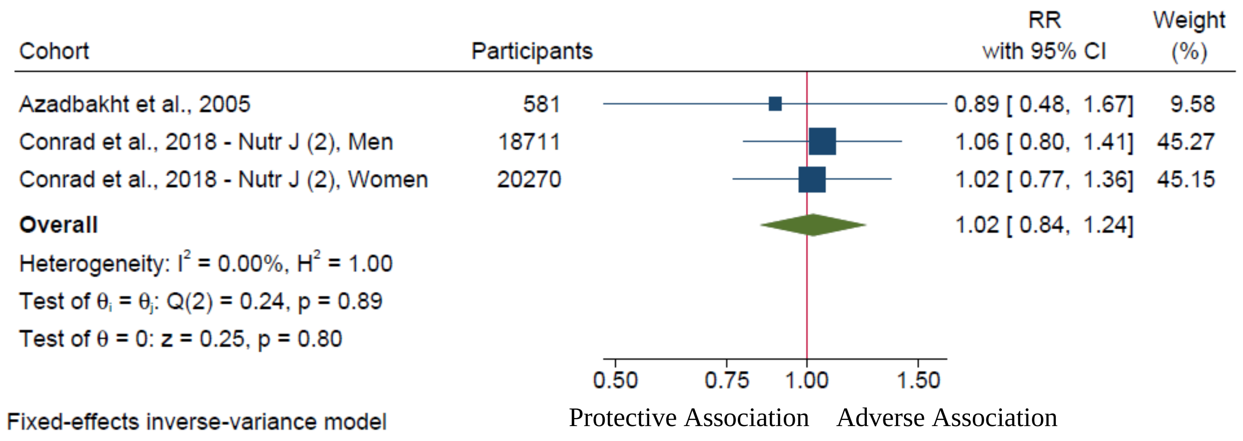

Diabetes in one study (Azadbakht et al., 2005) was defined at FBG≥7.0 mmol/L or 2HPG≥11.1 mmol/L.
2HPG = 2-hour postprandial glucose; CI = confidence interval; FBG = fasting blood glucose; RR = risk ratio.

Supplemental Figure S24. Forest plot of prospective cohort studies of the association between variety of vegetable and/or fruit intake and fasting glucose.

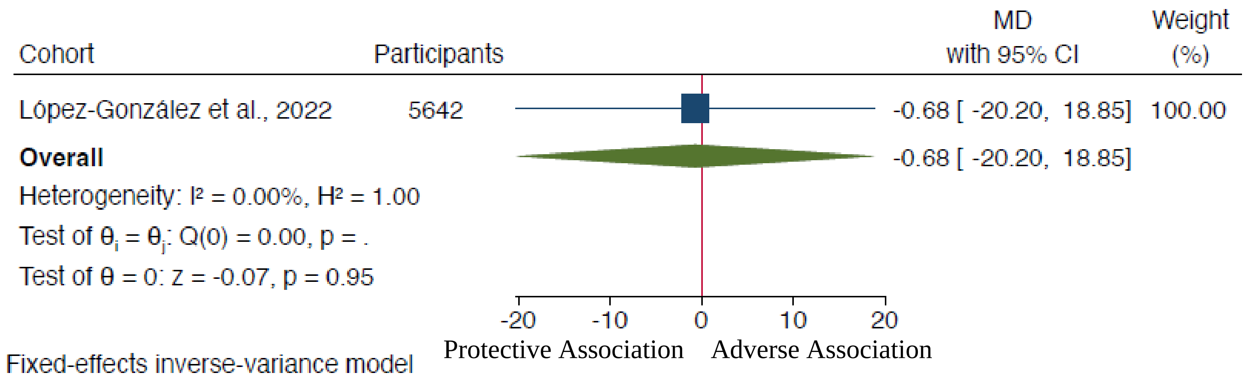

CI = confidence interval; MD = mean difference.

Supplemental Figure S25. Forest plot of prospective cohort studies of the association between variety of vegetable and/or fruit intake and hemoglobin A1c.

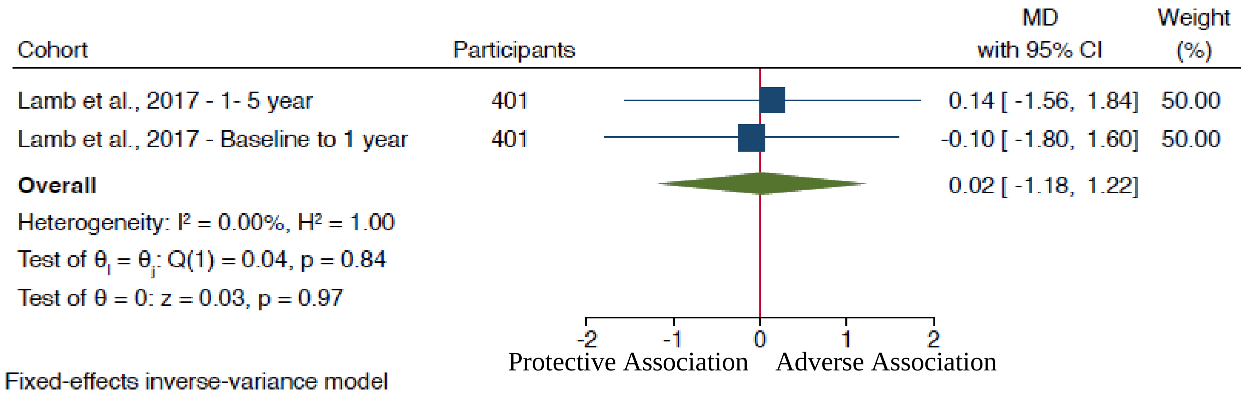

CI = confidence interval; MD = mean difference.

Supplemental Figure S26. Forest plot of prospective cohort studies of the association between variety of vegetable and/or fruit intake and CCMR.

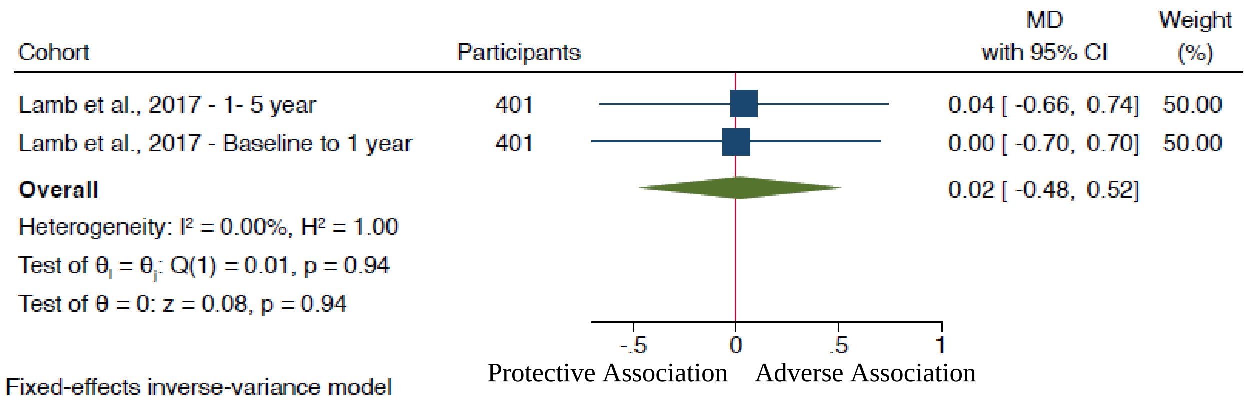

CCMR = Clustered cardiometabolic risk scores; CI = confidence interval; MD = mean difference.

Supplemental Figure S27. Forest plot of prospective cohort studies of the association between variety of vegetable and/or fruit intake and Framingham Risk Score.

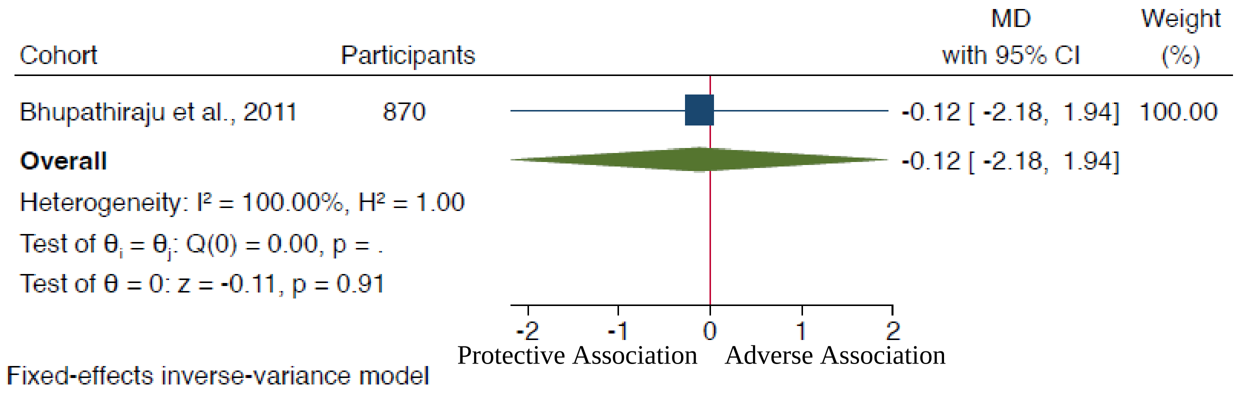

CI = confidence interval; MD = mean difference.

Supplemental Figure S28. Sensitivity analysis of the systematic removal of each cohort for the association between variety of vegetable and/or fruit intake and all-cause mortality in prospective cohorts.

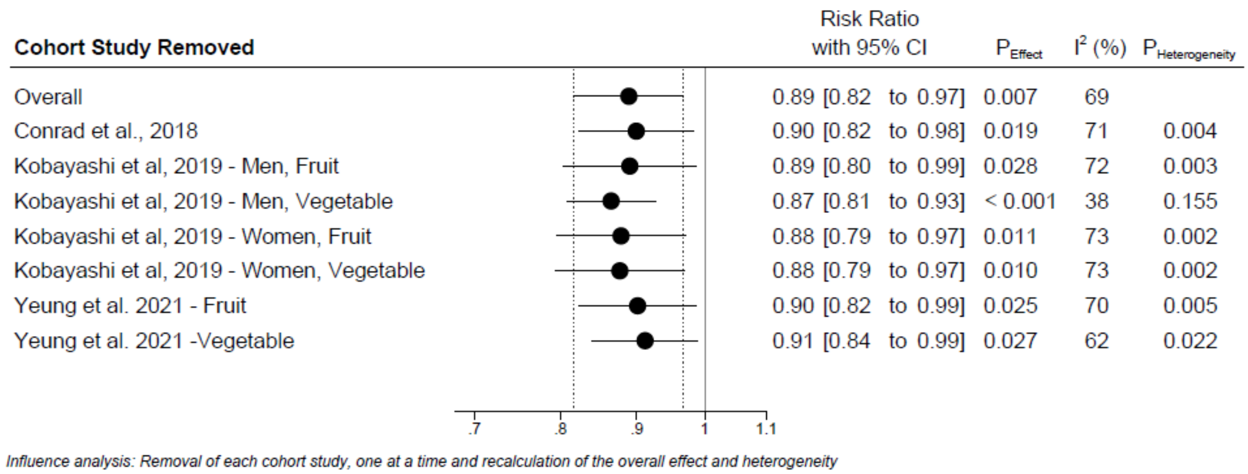

CI= confidence interval; I^2=heterogeneity

Supplemental Figure S29. Sensitivity analysis of the systematic removal of each cohort for the association between variety of vegetable and/or fruit intake and cardiovascular mortality in prospective cohorts.

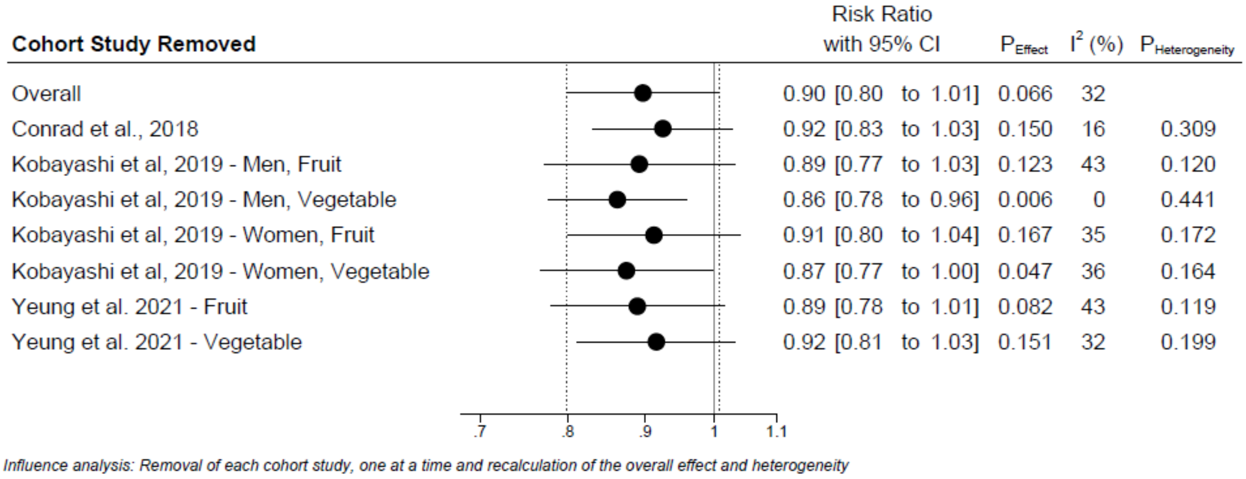

CI= confidence interval; I^2=heterogeneity

Supplemental Figure S30. Sensitivity analysis of the systematic removal of each cohort for the association between variety of vegetable and/or fruit intake and coronary heart disease incidence in prospective cohorts.

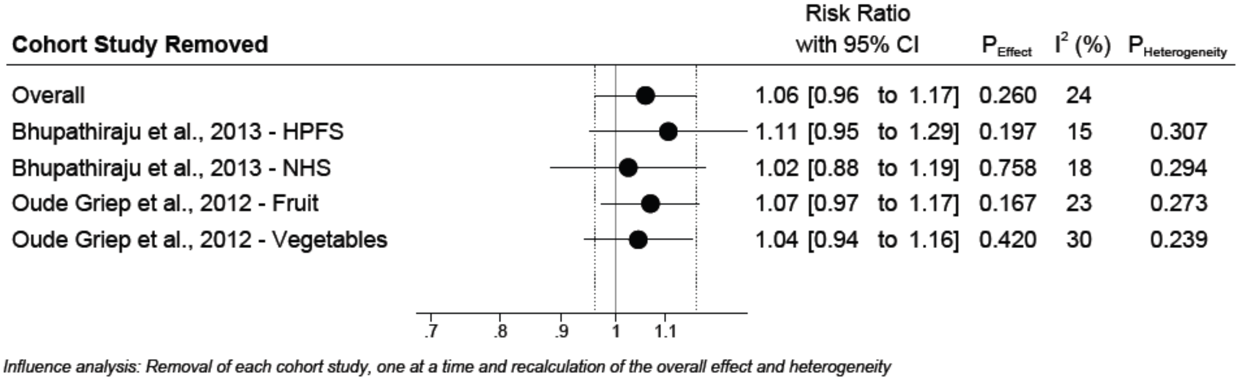

CI= confidence interval; I^2=heterogeneity

Supplemental Figure S31. Sensitivity analysis of the systematic removal of each cohort for the association between variety of vegetable and/or fruit intake and systolic blood pressure in prospective cohorts.

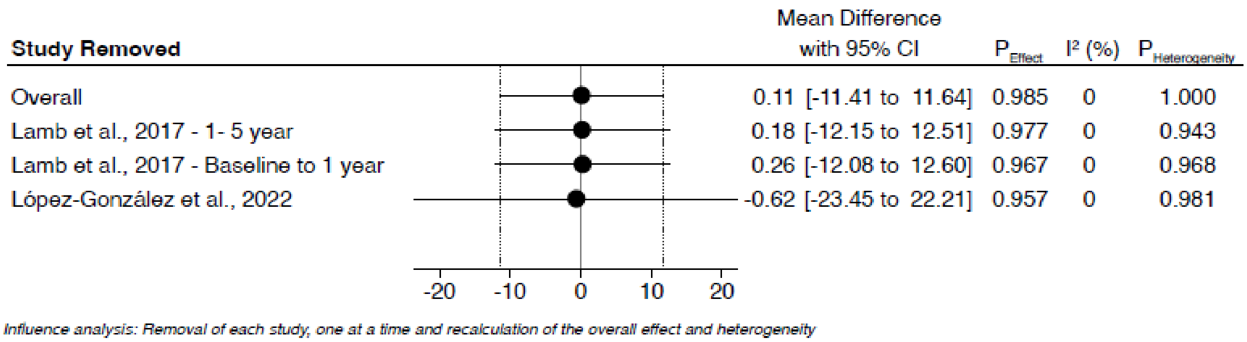

CI= confidence interval; I^2=heterogeneity

Supplemental Figure S32. Sensitivity analysis of the systematic removal of each cohort for the association between variety of vegetable and/or fruit intake and HDL-C in prospective cohorts.

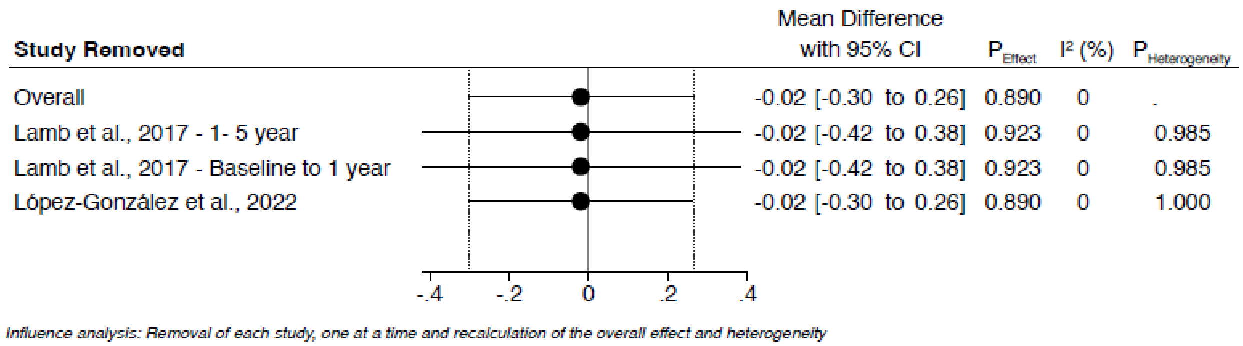

CI= confidence interval; HDL-C = high-density lipoprotein-cholesterol; I^2=heterogeneity

Supplemental Figure S33. Sensitivity analysis of the systematic removal of each cohort for the association between variety of vegetable and/or fruit intake and triglycerides in prospective cohorts.

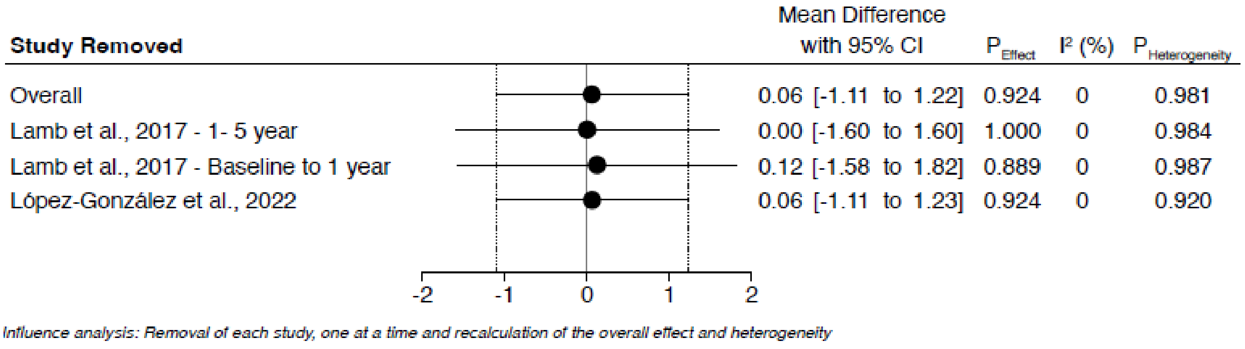

CI= confidence interval; I^2=heterogeneity

Supplemental Figure S34. Sensitivity analysis of the systematic removal of each cohort for the association between variety of vegetable and/or fruit intake and waist circumference in prospective cohorts.

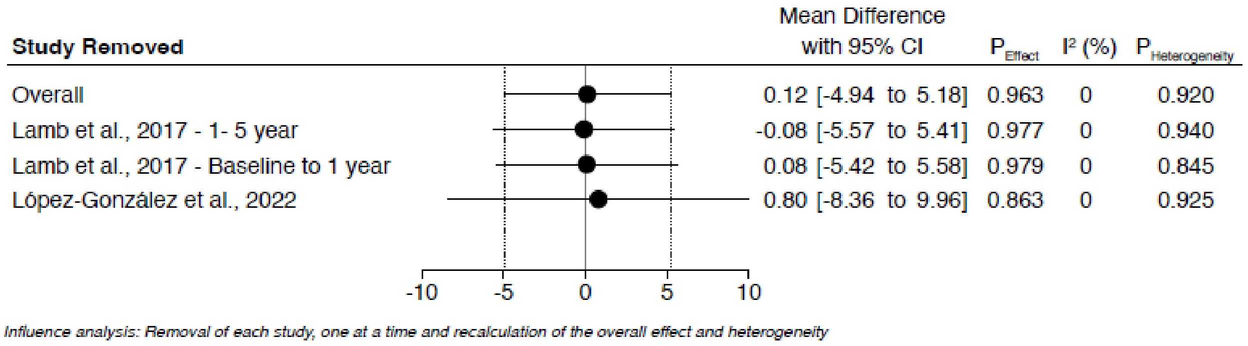

CI= confidence interval; I^2=heterogeneity

Supplemental Figure S35. Sensitivity analysis of the systematic removal of each cohort for the association between variety of vegetable and/or fruit intake and diabetes in cross-sectional studies.

Cohort Study Removed	Risk Ratio with 95% CI	P_{Effect}	I^2 (%)	$P_{Heterogeneity}$
Azadbakht et al., 2005	1.04 [0.85 to 1.27]	0.706	0	0.853
Conrad et al., 2018 - Nutr J (2), Men	1.00 [0.77 to 1.29]	0.979	0	0.704
Conrad et al., 2018 - Nutr J (2), Women	1.03 [0.79 to 1.34]	0.831	0	0.624

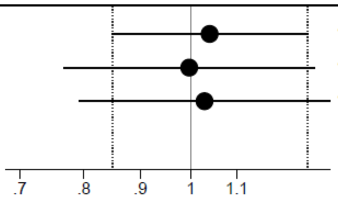

Influence analysis: Removal of each cohort study, one at a time and recalculation of the overall effect and heterogeneity

CI= confidence interval; I^2=heterogeneity

References

[1] L. Azadbakht, P. Mirmiran, A. Esmaillzadeh, F. Azizi, Dietary diversity score and cardiovascular risk factors in Tehranian adults, Public Health Nutr. 9 (2006) 728–736. https://doi.org/10.1079/phn2005887.

[2] M.A. Bernstein, K.L. Tucker, N.D. Ryan, E.F. O'neill, K.M. Clements, M.E. Nelson, W.J. Evans, M.A.F. Singh, Higher dietary variety is associated with better nutritional status in frail elderly people, J Am Diet Assoc. 102 (2002) 1096–1104.

[3] S.N. Bhupathiraju, K.L. Tucker, Greater variety in fruit and vegetable intake is associated with lower inflammation in Puerto Rican adults, American Journal of Clinical Nutrition. 93 (2011) 37–46. https://doi.org/10.3945/ajcn.2010.29913.

[4] Z. Conrad, S. Raatz, L. Jahns, Greater vegetable variety and amount are associated with lower prevalence of coronary heart disease: National Health and Nutrition Examination Survey, 1999-2014, Nutr J. 17 (2018). https://doi.org/10.1186/s12937-018-0376-4.

[5] Z. Conrad, J. Thomson, L. Jahns, Prospective analysis of vegetable amount and variety on the risk of all-cause and cause-specific mortality among US adults, 1999–2011, Nutrients. 10 (2018). https://doi.org/10.3390/nu10101377.

[6] S.N. Bhupathiraju, N.M. Wedick, A. Pan, J.E. Manson, K.M. Rexrode, W.C. Willett, E.B. Rimm, F.B. Hu, Quantity and variety in fruit and vegetable intake and risk of coronary heart disease1-3, American Journal of Clinical Nutrition. 98 (2013) 1514–1523. https://doi.org/10.3945/ajcn.113.066381.

[7] M. Kobayashi, S. Sasazuki, T. Shimazu, N. Sawada, T. Yamaji, M. Iwasaki, T. Mizoue, S. Tsugane, Association of dietary diversity with total mortality and major causes of mortality in the Japanese population: JPHC study, Eur J Clin Nutr. 74 (2020) 54–66. https://doi.org/10.1038/s41430-019-0416-y.

[8] M.J.E. Lamb, S.J. Griffin, S.J. Sharp, A.J.M. Cooper, Fruit and vegetable intake and cardiovascular risk factors in people with newly diagnosed type 2 diabetes, Eur J Clin Nutr. 71 (2017) 115–121. https://doi.org/10.1038/ejcn.2016.180.

[9] L. López-González, N. Becerra-Tomás, N. Babio, M.Á. Martínez-González, S.K. Nishi, D. Corella, A. Goday, D. Romaguera, J. Vioque, Á.M. Alonso-Gómez, J. Wärnberg, J.A. Martínez, L. Serra-Majem, R. Estruch, M.R. Bernal-López, J. Lapetra, X. Pintó, J.A. Tur, J. López-Miranda, A. Bueno-Cavanillas, M. Delgado-Rodríguez, P. Matía-Martín, L. Daimiel, V. Martín-Sánchez, J. Vidal, C. Vázquez, E. Ros, Z. Vázquez-Ruiz, F.M. Martín-Luján, J. V. Sorlí, O. Castañer, M.A. Zulet, L. Tojal-Sierra, R. Carabaño-Moral, J. Román-Maciá, E. Rayó, A. García-Ríos, R. Casas, A.M. Gómez-Pérez, J.M. Santos-Lozano, P. Buil-Cosiales, E.M. Asensio, C. Lassale, I. Abete, I. Salaverria-Lete, C. Sayón-Orea, H. Schröder, J. Salas-Salvadó, One-year changes in fruit and vegetable variety intake and cardiometabolic risk factors changes in a middle-aged Mediterranean population at high cardiovascular risk, Eur J Clin Nutr. (2022). https://doi.org/10.1038/s41430-022-01124-3.

[10] L.M. Oude Griep, W.M.M. Verschuren, D. Kromhout, M.C. Ocké, J.M. Geleijnse, Variety in fruit and vegetable consumption and 10-year incidence of CHD and stroke, Public Health Nutr. 15 (2012) 2280–2286. https://doi.org/10.1017/S1368980012000912.

[11] S. Yeung, Z. Zhu, R. Chan, T. Kwok, J. Woo, Prospective analysis of fruit and vegetable variety on health outcomes in community-dwelling Chinese older adults, J Nutr Health Aging. (2021).

[12] C.P. Cannon, M.A. Blazing, R.P. Giugliano, A. McCagg, J.A. White, P. Theroux, H. Darius, B.S. Lewis, T.O. Ophuis, J.W. Jukema, G.M. De Ferrari, W. Ruzyllo, P. De Lucca, K. Im, E.A. Bohula, C. Reist, S.D. Wiviott, A.M. Tershakovec, T.A. Musliner, E. Braunwald, R.M. Califf, Ezetimibe Added to Statin Therapy after Acute Coronary Syndromes, New England Journal of Medicine. 372 (2015) 2387–2397. https://doi.org/10.1056/nejmoa1410489.

[13] Cholesterol Treatment Trialists´ (CTT) Collaboration, Efficacy and safety of more intensive lowering of LDL cholesterol: a meta-analysis of data from 170 000 participants in 26 randomised trials, The Lancet. 376 (2010) 1670–1681. https://doi.org/10.1016/S0140-6736(10)61350-5.

[14] F.L.J. Visseren, F. Mach, Y.M. Smulders, D. Carballo, K.C. Koskinas, M. Bäck, A. Benetos, A. Biffi, J.-M. Boavida, D. Capodanno, B. Cosyns, C. Crawford, C.H. Davos, I. Desormais, E. Di Angelantonio, O.H. Franco, S. Halvorsen, F.D.R. Hobbs, M. Hollander, E.A. Jankowska, M. Michal, S. Sacco, N. Sattar, L. Tokgozoglu, S. Tonstad, K.P. Tsioufis, I. van Dis, I.C. van Gelder, C. Wanner, B. Williams, G. De Backer, V. Regitz-Zagrosek, A.H. Aamodt, M. Abdelhamid, V. Aboyans, C. Albus, R. Asteggiano, M. Bäck, M.A. Borger, C. Brotons, J. Čelutkienė, R. Cifkova, M. Cikes, F. Cosentino, N. Dagres, T. De Backer, D. De Bacquer, V. Delgado, H. Den Ruijter, P. Dendale, H. Drexel, V. Falk, L. Fauchier, B.A. Ference, J. Ferrières, M. Ferrini, M. Fisher, D. Fliser, Z. Fras, D. Gaita, S. Giampaoli, S. Gielen, I. Graham, C. Jennings, T. Jorgensen, A. Kautzky-Willer, M. Kavousi, W. Koenig, A. Konradi, D. Kotecha, U. Landmesser, M. Lettino, B.S. Lewis, A. Linhart, M.-L. Løchen, K. Makrilakis, G. Mancia, P. Marques-Vidal, J.W. McEvoy, P. McGreavy, B. Merkely, L. Neubeck, J.C. Nielsen, J. Perk, S.E. Petersen, A.S. Petronio, M. Piepoli, N.G. Pogosova, E.I.B. Prescott, K.K. Ray, Z. Reiner, D.J. Richter, L. Rydén, E. Shlyakhto, M. Sitges, M. Sousa-Uva, I. Sudano, M. Tiberi, R.M. Touyz, A. Ungar, W.M.M. Verschuren, O. Wiklund, D. Wood, J.L. Zamorano, Y.M. Smulders, D. Carballo, K.C. Koskinas, M. Bäck, A. Benetos, A. Biffi, J.-M. Boavida, D. Capodanno, B. Cosyns, C.A. Crawford, C.H. Davos, I. Desormais, E. Di Angelantonio, O.H. Franco Duran, S. Halvorsen, F.D. Richard Hobbs, M. Hollander, E.A. Jankowska, M. Michal, S. Sacco, N. Sattar, L. Tokgozoglu, S. Tonstad, K.P. Tsioufis, I. van Dis, I.C. van Gelder, C. Wanner, B. Williams, 2021 ESC Guidelines on cardiovascular disease prevention in clinical practice, Eur Heart J. 42 (2021) 3227–3337. https://doi.org/10.1093/eurheartj/ehab484.

[15] Prospective Studies Collaboration, Age-specific relevance of usual blood pressure to vascular mortality: a meta-analysis of individual data for one million adults in 61 prospective studies, The Lancet. 360 (2002) 1903–1913. https://doi.org/10.1016/S0140-6736(02)11911-8.

[16] E. Medicines Agency, Committee for Medicinal Products for Human Use (CHMP) 3 Guideline on clinical investigation of medicinal products in, 2018. www.ema.europa.eu/contact.

[17] R. Borg, J.C. Kuenen, B. Carstensen, H. Zheng, D.M. Nathan, R.J. Heine, J. Nerup, K. Borch-Johnsen, D.R. Witte, Associations between features of glucose exposure and A1C: The A1C-Derived Average Glucose (ADAG) study, Diabetes. 59 (2010) 1585–1590. https://doi.org/10.2337/db09-1774.

[18] L. Ge, B. Sadeghirad, G.D.C. Ball, B.R. da Costa, C.L. Hitchcock, A. Svendrovski, R. Kiflen, K. Quadri, H.Y. Kwon, M. Karamouzian, T. Adams-Webber, W. Ahmed, S. Damanhoury, D. Zeraatkar, A. Nikolakopoulou, R.T. Tsuyuki, J. Tian, K. Yang, G.H.

Guyatt, B.C. Johnston, Comparison of dietary macronutrient patterns of 14 popular named dietary programmes for weight and cardiovascular risk factor reduction in adults: systematic review and network meta-analysis of randomised trials, BMJ. (2020) m696. https://doi.org/10.1136/bmj.m696.
[19] B.C. Johnston, S. Kanters, K. Bandayrel, P. Wu, F. Naji, R.A. Siemieniuk, G.D.C. Ball, J.W. Busse, K. Thorlund, G. Guyatt, J.P. Jansen, E.J. Mills, Comparison of weight loss among named diet programs in overweight and obese adults: A meta-analysis, JAMA. 312 (2014) 923–933. https://doi.org/10.1001/jama.2014.10397.
[20] Reynolds Risk Score: Calculating Heart and Stroke Risk for Women and Men, (n.d.). http://www.reynoldsriskscore.org/ (accessed May 27, 2023).
[21] P.M. Ridker, N.P. Paynter, N. Rifai, J.M. Gaziano, N.R. Cook, C-reactive protein and parental history improve global cardiovascular risk prediction: The Reynolds risk score for men, Circulation. 118 (2008) 2243–2251. https://doi.org/10.1161/CIRCULATIONAHA.108.814251.
[22] P.M. Ridker, J.E. Buring, N. Rifai, N.R. Cook, Development and Validation of Improved Algorithms for the Assessment of Global Cardiovascular Risk in Women The Reynolds Risk Score, n.d. https://jamanetwork.com/.
[23] Canadian Cardiovascular Society, Framingham Risk Score (FRS), (2017). https://ccs.ca/app/uploads/2020/12/FRS_eng_2017_fnl1.pdf (accessed May 27, 2023).
[24] N. Santesso, C. Glenton, P. Dahm, P. Garner, E.A. Akl, B. Alper, R. Brignardello-Petersen, A. Carrasco-Labra, H. De Beer, M. Hultcrantz, T. Kuijpers, J. Meerpohl, R. Morgan, R. Mustafa, N. Skoetz, S. Sultan, C. Wiysonge, G. Guyatt, H.J. Schünemann, GRADE Working Group, GRADE guidelines 26: informative statements to communicate the findings of systematic reviews of interventions., J Clin Epidemiol. 119 (2020) 126–135. https://doi.org/10.1016/j.jclinepi.2019.10.014.
[25] H.J. Schünemann, W. Wiercioch, J. Brozek, I. Etxeandia-Ikobaltzeta, R.A. Mustafa, V. Manja, R. Brignardello-Petersen, I. Neumann, M. Falavigna, W. Alhazzani, N. Santesso, Y. Zhang, J.J. Meerpohl, R.L. Morgan, B. Rochwerg, A. Darzi, M.X. Rojas, A. Carrasco-Labra, Y. Adi, Z. AlRayees, J. Riva, C. Bollig, A. Moore, J.J. Yepes-Nuñez, C. Cuello, R. Waziry, E.A. Akl, GRADE Evidence to Decision (EtD) frameworks for adoption, adaptation, and de novo development of trustworthy recommendations: GRADE-ADOLOPMENT, J Clin Epidemiol. 81 (2017) 101–110. https://doi.org/10.1016/j.jclinepi.2016.09.009.
[26] H. Balshem, M. Helfand, H.J. Schünemann, A.D. Oxman, R. Kunz, J. Brozek, G.E. Vist, Y. Falck-Ytter, J. Meerpohl, S. Norris, G.H. Guyatt, GRADE guidelines: 3. Rating the quality of evidence, J Clin Epidemiol. 64 (2011) 401–406. https://doi.org/10.1016/j.jclinepi.2010.07.015.
